# Diverse, novel mycoviruses coinfecting the phytopathogenic fungus *Corynespora cassiicola* from *Sesamum indicum*

**DOI:** 10.3389/fcimb.2025.1704628

**Published:** 2026-01-05

**Authors:** Mingming Liu, Yunxia Ni, Jing Wang, Xintao Liu, Hui Zhao, Xinbei Zhao, Wenqing Yan, Hongyan Liu, Baoming Tian, Hongmei Miao

**Affiliations:** 1 Institute of Plant Protection, Henan Academy of Agricultural Sciences, Key Laboratory of Integrated Crop Pests Management on Crops in Southern Region of North China, Ministry of Agriculture and Rural Affairs, Henan Key Laboratory of Agricultural Pest Monitoring and Control, Postgraduate Teaching and Research Base of Zhengzhou University, Zhengzhou, Henan, China; 2 School of Agricultural Sciences, Zhengzhou University, Zhengzhou, Henan, China; 3 Henan Sesame Research Center, Henan Academy of Agricultural Sciences, Zhengzhou, Henan, China

**Keywords:** mycoviruses, *Corynespora cassiicola*, *Corynespora* leaf spot, virus diversity, biological control

## Abstract

**Introduction:**

Corynespora leaf spot caused by *Corynespora cassiicola* is one of the most serious foliar diseases of sesame. Mycoviruses can infect various groups of fungi and have certain potential biocontrol value. However, the number of mycovirus reported in *C. cassiicola* is relatively scarce.

**Methods:**

Seven strains of *C. cassiicola* isolated from *Sesamum indicum* were subjected to metatranscriptomic sequencing. Viral diversity was further assessed and validated through RT-PCR, followed by sequence alignment and phylogenetic analysis.

**Results:**

A total of 19 viruses were identified across the seven strains, distributed among 12 families. +ssRNA viruses were identified, belonging to *Botourmiaviridae* (four viruses), *Deltaflexiviridae* (one virus), *Fusariviridae* (two viruses), *Narnaviridae* (two viruses), *Ambiguiviridae* (two viruses), and *Potyviridae* (one virus). −ssRNA viruses were assigned Mymonaviridaeto (two viruses), and Mycophioviridae (one virus). dsRNA viruses were found, including *Chrysoviridae* (one virus), *Partitiviridae* (one virus), *Totiviridae* (one virus), and *Polymycoviridae* (one virus).

**Discussion:**

This study uses metatranscriptome technology to expand our understanding of the diversity of mycoviruses in *C. cassiicola* and provides a rich resource for future studies on mycovirus diversity and taxonomy.

## 1 Introduction

Viruses are among the most abundant and diverse biological entities on Earth, capable of infecting various life forms, including animals (Ji et al., 2024), plants (Wu et al., 2024), bacteria (Weinbauer, 2004), protists (La Scola, 2014), and fungi (Ghabrial and Suzuki, 2009). However, most virus-related studies have primarily focused on plant and animal viruses of economic importance, often neglecting other significant viruses such as fungal viruses (mycoviruses). Mycoviruses can infect fungi, and their classification is based on factors such as host range, genome structures, and the phylogenetic relationships of viral proteins (Kondo et al., 2022). Most mycoviruses have double-stranded RNA (dsRNA) or positive-sense single-stranded RNA (+ssRNA) genomes; additionally, some mycoviruses with linear negative-sense single-stranded RNA (−ssRNA) (Liu et al., 2014), single-stranded DNA (ssDNA) (Yu et al., 2010), or circular single-stranded RNA genomes have also been discovered (Schiwek et al., 2024).

In the 1960s, M. Hollings first discovered mycoviruses in *Agaricus bisporus*, an edible cultivated mushroom belonging to the *Basidiomycota* (Ghabrial, 1998). Thereafter, L. F. Ellis reported the presence of mycoviruses in *Penicillium stoloniferum* of the *Ascomycota* (Kotta-Loizou, 2021). In recent years, advances in high-throughput next-generation sequencing (NGS) and bioinformatics have revolutionized the discovery of viruses in various organisms, including fungi. Consequently, the known diversity of mycoviruses has increased rapidly, with some possessing unprecedented genomes (Mu et al., 2021; Ruiz-Padilla et al., 2021; Lu et al., 2024; Zhang et al., 2024). As novel mycoviruses that cannot be classified into existing taxa are discovered, the classification system of mycoviruses is constantly being improved.

Most mycoviruses cause no obvious symptoms in their hosts (Ghabrial and Suzuki, 2009). However, some mycoviruses can inhibit fungal growth, sporulation, virulence, and mycotoxin production and may also cause deformities in fruiting bodies, thereby reducing the virulence of plant pathogenic fungi (Xie and Jiang, 2014; García-Pedrajas et al., 2019). Hypovirulence-associated mycoviruses of biocontrol interest, such as Cryphonectria hypovirus 1 (CHV-1), are used for the biological control of chestnut blight (Feau et al., 2014; Rigling and Prospero, 2018). With the deepening of research, more and more mycoviruses that can cause phenotypic changes in fungi have been identified. For example, Sclerotinia sclerotiorum hypovirulence-associated DNA virus 1 (SsHADV-1) can effectively control Sclerotinia stem rot of rapeseed (Yu et al., 2013). In addition, Pestalotiopsis theae chrysovirus 1 (PtCV1) can transform its host fungus into a non-pathogenic endophytic fungus (Zhou et al., 2021b). Diaporthe sojae circular DNA virus 1 (DsCDV1) can also significantly reduce the pathogenicity of the fungus (Wang et al., 2024). In addition, the virus-induced gene silencing (VIGS) vector of Fusarium graminearum gemytripvirus 1 (FgGMTV1) can successfully convert the pathogenic fungus causing Fusarium head blight (FHB) in cereal crops into hypovirulent strains (Zhang et al., 2023). Consequently, exploring mycovirus diversity is a fruitful path for discovering new biological control agents.

*Corynespora cassiicola* is an ascomycete with extensive genetic diversity, distributed across tropical and subtropical regions worldwide (Dixon et al., 2009; Sierra-Orozco et al., 2023). It can infect more than 530 species of monocotyledonous and dicotyledonous plants, including numerous important crops (Sumabat et al., 2018). In addition to plants, this fungus can also be isolated from nematode cysts and can cause opportunistic infections in immunocompromised human patients (Arango-Franco et al., 2018). In China, it is a severe pathogen causing leaf spot in major sesame-producing areas. Infections of the flowers, fruits, and roots of sesame lead to leaf withering and abscission, impair photosynthesis, and consequently cause seed wilting and reduced oil content (Jia et al., 2024). However, despite its significant impact on agriculture, reports of mycoviruses infecting *C. cassiicola* remain limited. Prior to this study, only three viruses had been reported in this fungus: one belonging to *Fusariviridae* (Liu et al., 2022a), one to *Totiviridae* (Liu et al., 2022b), and a dsRNA with uncertain classification (Wang et al., 2020).

This study conducted a metatranscriptomic survey to analyze the viral diversity of *C. cassiicola* strains from sesame in Henan Province, China. We identified 19 RNA viruses spanning 12 distinct families and confirmed their presence in each isolate via RT-PCR. While the complete genomes of Corynespora cassiicola fusarivirus 1 (CcFV1) and Corynespora cassiicola victorivirus 1 (CcVV1) were previously reported (Liu et al., 2022a, 2022b), our work significantly expands the known virome of this pathogen. These findings provide a foundational resource for understanding mycovirus diversity and evolution in *C. cassiicola.*

## 2 Materials and methods

### 2.1 Fungal growth conditions and microscopic observation

In this study, seven *C. cassiicola* strains isolated from sesame leaf spot lesions, selected for their abnormal morphology (the colonies are whitish and grow slowly) or the presence of detectable dsRNA by cellulose chromatography, were chosen from a collection of 54 isolates originating from diverse geographic regions (**Supplementary Table 1**). Sterilized coverslips were placed on the edge of potato dextrose agar (PDA) Petri dishes, and then 6-mm-diameter fungal cakes were inoculated in the center of the PDA plates. The plates were incubated at 28°C in the dark for 7 days. When the mycelia grew onto the coverslips, the coverslips were picked up from the edge with sterile tweezers, and the side with mycelia was placed on a glass slide. The morphology of the mycelial tips was observed under a microscope and photographed (Wang et al., 2021).

### 2.2 Determination of growth rate, pathogenicity, and sporulation capacity of seven *C. cassiicola* strains

An 8-mm-diameter mycelial plug was excised from the edge of a fungal colony and placed onto the center of a PDA plate. The plate was incubated at a constant temperature of 28°C under an alternating light condition (12 h light/12 h dark). Each treatment were replicated three times. After 8 days of incubation, the colony diameter was measured to calculate the growth rate of the *Corynespora* strains.

*Corynespora* strains were cultured on PDA at 28°C for 8 days under a 12-h light/12-h dark cycle. 6-mm-diameter mycelial discs were punched out from the colonies, and each disc was transferred into a 1.5-mL sterile centrifuge tube. 1 mL of sterile water was added to the tube and vortexed thoroughly for 1 min to suspend the spores, and then a small amount of the spore suspension was drawn and placed on a hemocytometer. Each strain was tested four times to determine the sporulation.

Sterilized filter paper was spread evenly at the bottom of a tray. The second pair of true leaves was taken from sesame plants at the three-leaf-one-heart stage and placed in the tray, and the petioles were wrapped with cotton balls moistened with sterile water to prevent leaf dehydration. Then, a 5-mm-diameter puncher was used to cut out fungal cakes, which were placed on the center of the leaves with the mycelium facing downward and covered with plastic wrap to maintain moisture. Each strain was set with six replicates. The samples were incubated at 28°C under a photoperiod of 16 h light/8 h dark. On the 6th day after inoculation, the size of leaf spots was determined using the cross method to assess the pathogenicity of the *Corynespora* strains.

All phenotypic assays, including growth rate, sporulation, and pathogenicity, were performed with at least three independent biological replicates. Error bars indicate ± standard deviation (SD). The significance of differences among the different fungal isolates was determined by one-way analysis of variance (ANOVA) followed by Duncan’s multiple range test. Prior to ANOVA, the assumptions of normality and homogeneity of variances were verified using the Shapiro–Wilk test and Levene’s test, respectively. All statistical analyses were conducted using SPSS (version 26.0), and *p* < 0.05 was considered statistically significant. The different letters above each column indicated that the corresponding values were significantly different at the *p* < 0.05 level.

### 2.3 RNA extraction and high-throughput sequencing

Mycelial agar blocks of seven *C. cassiicola* isolates were inoculated on PDA plates and cultured at 28°C in the dark for 7–8 days. Mycelia were collected from each Petri dish using a sterile spoon. Subsequently, total RNA was extracted from approximately 1.0 g of mycelia using the RNAiso Kit (TaKaRa, Dalian, China). The total RNA digested with DNase from the seven isolates was mixed into one sample and sent to Shanghai Biotechnology Corporation for high-throughput sequencing on the Illumina HiSeq 2500 platform. Total RNA was checked for a RIN number to inspect RNA integrity by an Agilent Bioanalyzer 2100. Qualified total RNA was further purified by RNAClean XP Kit (Cat. A63987, Beckman Coulter, Inc., Kraemer Boulevard Brea, CA, USA). Through library construction and quality inspection, cluster generation, and paired-end sequencing using the paired-end program, a total of 10-G data volume was obtained, with the proportion of bases with quality scores greater than 20 (Q20) in each direction not less than 85%. Raw reads with low overall and terminal quality and those containing sequencing primers were removed to obtain clean reads. Primary unigenes were obtained by *de novo* assembly, and the CAP3 EST assembly software was used for the second assembly of primary unigenes to get a set of final unigene sequences. The obtained final unigene sequences were subjected to BLASTx alignment with the NR protein database, and contig sequences whose first matched hit was a virus were identified as potential viral sequences.

### 2.4 Putative mycovirus sequence confirmation

cDNA of *C. cassiicola* isolates was synthesized following the instructions provided with the PrimeScript II™ 1st Strand cDNA Synthesis Kit (TaKaRa, Dalian, China). To confirm the presence of the newly discovered virus in each isolate, RT-PCR was performed using virus-specific primers to detect the specific amplification product of each virus (**Supplementary Table S4**). The amplification product contained a partial fragment of the RNA-dependent RNA polymerase (RdRp) gene. The total volume of the RT-PCR reaction was 25 μL, which included 9.5 μL of deionized water, 12.5 μL of Premix Taq™, 1 μL each of forward and reverse primers at a concentration of 10 μM, and 1 μL of cDNA template. The annealing temperature and extension time were determined according to each primer and product size. The RT-PCR products were subjected to agarose gel electrophoresis. Products with target bands were sent to Sangon Biotech for sequencing and verification.

### 2.5 Mycoviral genome analysis and phylogenetic analysis

The assembled contigs were compared against the NCBI non-redundant database (https://blast.ncbi.nlm.nih.gov/Blast.cgi) via BLASTX and BLASTn analyses, based on the translated amino acid sequences and nucleotide sequences, respectively. Sequence alignment was performed using the CLUSTALX program, and a phylogenetic tree was constructed with the MEGA program (version 7.0) using the neighbor-joining method, with the bootstrap value set to 1,000 replicates. The evolutionary distances were computed using the Poisson correction method and are in the units of the number of amino acid substitutions per site.

## 3 Results

### 3.1 Biological characteristics of seven *C. cassiicola*

**Figure 1:**
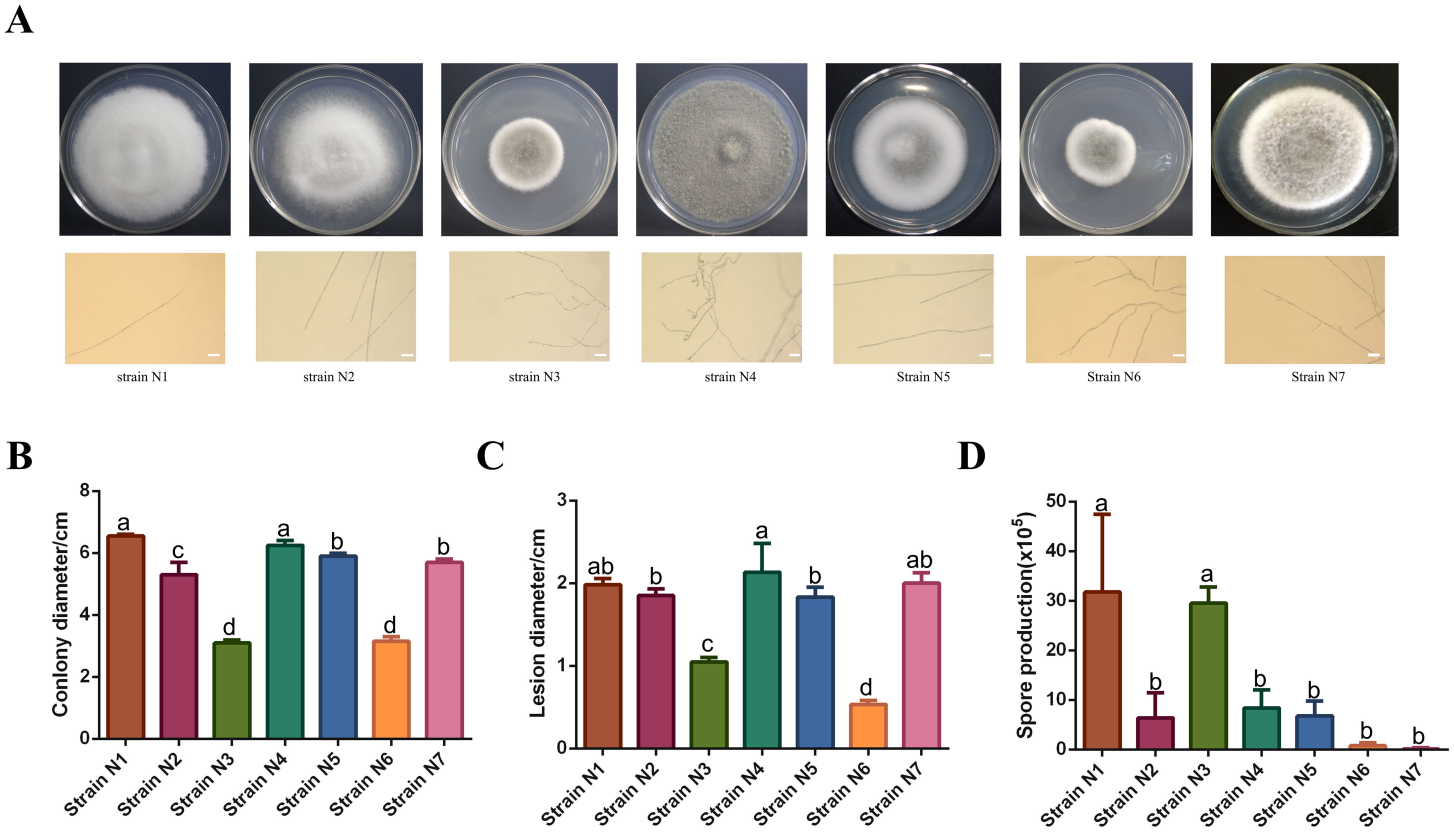
Statistical analysis of the biological characteristics of seven Corynespora strains. **(A)** Colony and hyphal tip morphology of 7 *C. cassiicola* strains. Scale bar is 50 μm. Statistical analysis of the growth rate **(B)**, pathogenicity **(C)**, and spore production **(D)** of the strains. Error bars indicate ± standard deviation (SD). The number of biological replicates for each assay is provided in the respective Methods section. Different lowercase letters above the bars indicate significant differences (*p* < 0.05) as determined by Duncan’s multiple range test.

Morphological observation was conducted on the seven *C. cassiicola* strains that were sent for metatranscriptome sequencing (**Figure 1**), revealing that the strains exhibited diverse morphologies and colors. Four strains, namely, strain N1, strain N2, strain N5, and strain N7, had white colonies and produced long, unbranched hyphae. Strain N4 showed a dark-brown colony with dense aerial mycelia and branched mycelial tips, and the average colony diameter reached 5.7 cm on the 8th day, showing a relatively faster growth rate compared with other strains. Strain N3 and strain N6 grew slowly; the former had relatively thin and weak mycelial tips, while the latter had curved and highly branched mycelial tips.

To clarify the biological characteristics of seven *C. cassiicola* strains, we statistically analyzed the growth rate, pathogenicity, and sporulation quantity of each strain. The results showed that the growth rates of strain N3 and strain N6 were the lowest, while those of strain N1 and strain N4 were the highest (**Figure 1B**). In addition, the lesion diameters on sesame leaves infected by strain N3 and strain N6 were the smallest, which were significantly different from those infected by strain N1, strain N2, strain N4, strain N5, and strain N7 (**Figure 1C**). However, strain N3, which had a lower growth rate and pathogenicity and exhibited a significantly higher spore production compared with the other strains (**Figure 1D**).

### 3.2 Metatranscriptomic identification of mycoviruses infecting isolates of *C. cassiicola*

Based on the metatranscriptome sequencing, the number of raw reads obtained was 6.1×10^7^. After filtering out unqualified reads, the final number of clean reads available for data analysis was 6.0×10^7^, accounting for 98.93%. Through *de novo* initial assembly, 33,373 primary unigenes were obtained, with an average length of 725 bp and a GC content of 53.5%. The CAP3 EST assembly software was used for the second assembly of the primary unigenes, and finally 31,660 contig sequences were obtained, the longest reaching 2.0×10^4^ bp, and the GC content was 53.49%. The final unigene sequences were subjected to BLASTx alignment with the NR database, and 20,537 sequences were annotated, with an annotation rate of 64.87%. Among them, 72 contigs showed high similarity to viruses in the NCBI database (**Supplementary Table 2**).

To verify whether the 72 viral sequences obtained from metatranscriptome sequencing truly exist in the tested strains, RT-PCR was performed on the strain cDNAs, followed by agarose gel electrophoresis detection. It was finally confirmed that 25 viral sequences truly exist in different strains (**Supplementary Figure 2**). There are significant differences in the types and quantities of viruses carried by different strains. Strain N3 carried nine viral fragments, belonging to six different virus families; strains N1, N2, and N5 all carried six viral fragments, belonging to different viral families respectively. In contrast, strain N6 only carried one viral fragment, which belonged to *Potyviridae* families (**Supplementary Table 3**).

A classification summary of the 25 viral sequences (**Table 1**) showed that the viruses carried by the tested strains belonged to 12 different families. The majority were (+)ssRNA viruses (63%), followed by dsRNA (21%) and (−)ssRNA viruses (16%). No ssDNA viruses or retroviruses carried by *C. cassiicola* were found. Among them, the *Botourmiaviridae* family has the largest number of viruses, with four viruses, accounting for 21%, while *Deltaflexiviridae*, *Potyviridae*, Mycophioviridae, *Chrysoviridae*, *Partitiviridae*, *Totiviridae*, and *Polymycoviridae* each contain only one virus, accounting for 5% (**Supplementary Tables 5**, **6**;**Supplementary Figure 3**).

**Table 1:** List of viruses discovered in *Corynespora cassiicola* from *Sesamum indicum*.

Contig number	Accession numbers	Length	Name of putative viruses	Cover	E value	Identity	Domain	BLASTx first hit	Family	Genus	Genome type
Contig181	PX316551	2,653	Corynespora cassiicola ourmia-like virus 1 (CcOLV 1)	78%	0	60.03%	RdRp	Botourmiaviridae sp. (UJQ91941.1)	*Botourmiaviridae*	*Penoulivirus*	ssRNA(+)
First_Contig27	PX316552	1,409	Corynespora cassiicola ourmia-like virus 2 (CcOLV 2)	97%	2E-137	51.30%	RdRp	Botourmiaviridae sp. (WAK77777.1)	*Botourmiaviridae*	*Penoulivirus*	ssRNA(+)
Contig163	PX316553	1,285	Corynespora cassiicola ourmia-like virus 3 (CcOLV 3)	87%	3E-123	56.65%	RdRp	Diplodia seriata botourmiavirus 1 (UOK20173.1)	*Botourmiaviridae*	*Magoulivirus*	ssRNA(+)
Contig532	PX316554	2,129	Corynespora cassiicola ourmia-like virus 7 (CcOLV 4)	90%	0	57.48%	RdRp	Botourmiaviridae sp. (WAK77879.1)	*Botourmiaviridae*	*Penoulivirus*	ssRNA(+)
Contig185	PX316555	7,931	Corynespora cassiicola deltaflexivirus 1 (CcDv1)	76%	0	99.57%	RdRp	Sesame deltaflexivirus 1 (QQG34641.1)	*Deltaflexiviridae*	*Deltaflexivirus*	ssRNA(+)
Contig2308	PX316556	6,422	Corynespora cassiicola fusarivirus 1 (CcFV1)	71%	0	100%	RdRp	Corynespora cassiicola fusarivirus 1 (ULO04603.1)	*Fusariviridae*	*-*	ssRNA(+)
Contig3361	PX316557	5,944	Corynespora cassiicola fusarivirus 2 (CcFV2)	69%	0	98.32%	RdRp	Corynespora cassiicola fusarivirus 1 (ULO04603.1)	*Fusariviridae*	*-*	ssRNA(+)
Contig1327	UIW13880	2,537	Corynespora cassiicola narnavirus 1(CcNv1)	57%	1E−55	33.14%	RdRp	Rhizoctonia solani narnavirus 13 (UIW13880.1)	*Narnaviridae*	*Narnavirus*	ssRNA (+)
Contig1035	PX316558	2,129	Corynespora cassiicola narnavirus 7(CcNv2)	96%	2E−137	38.91%	RdRp	Plasmopara viticola lesion associated narnavirus 17 (QIR30296.1)	*Narnaviridae*	*Narnavirus*	ssRNA (+)
First_Contig11	PX316559	3,514	Corynespora cassiicola ambiguivirus 1 (CcAv1)	42%	0	61.45%	RdRp	Alternaria dianthicola umbra-like virus 1 (UYZ32451.1)	Ambiguiviridae	*-*	ssRNA (+)
Contig333	PX316560	3,601	Corynespora cassiicola ambiguivirus 2 (CcAv2)	40%	0	71.52%	RdRp	Phoma matteucciicola RNA virus 1 (QNC69246.1)	Ambiguiviridae	*-*	ssRNA(+)
First_Contig281	PX316561	3,619	Corynespora cassiicola potyvirus 1 (CcPov1)	100%	0	84.25%	Polypeptide	Wisteria vein mosaic virus (BDS00297.1)	*Potyviridae*	*Potyvirus*	ssRNA (+)
Contig27	PX316562	6,519	Corynespora cassiicola mymonavirus 1 (CcMV1)	88%	0	70.25%	RdRp	Grapevine wood holobiome associated mononegaambi virus 2 (XLV09868.1)	*Mymonaviridae*	*-*	ssRNA(−)
Contig28	PX316563	6,619	Corynespora cassiicola mymonavirus 2 (CcMV2)	88%	0	67.08%	RdRp	Grapevine wood holobiome associated mononegaambi virus 2 (XLV09868.1)	*Mymonaviridae*	*-*	ssRNA(−)
Contig47	PX316564	7,265	Corynespora cassiicola negative-stranded RNA virus 1 (CcRV1)	97%	0	76.44%	RdRp	Grapevine wood holobiome associated mycoophiovirus 1 (XNS37001.1)	Mycophioviridae	*-*	ssRNA(−)
Contig829	PX316565	3,523	Corynespora cassiicola chrysovirus 1 RNA1 (CcChv1RNA1)	94%	0	59.03%	RdRp	Diplodia seriata chrysovirus 1 (UOK20161.1)	*Chrysoviridae*	*Alphachrysovirus*	dsRNA
Contig30	PX316566	3,050	Corynespora cassiicola chrysovirus 1 RNA2 (CcChv1RNA2)	94%	0	35.35%	capsid protein	Diplodia seriata chrysovirus 1 (UOK20162.1)	*Chrysoviridae*	*Alphachrysovirus*	dsRNA
Contig895	PX316567	3,059	Corynespora cassiicola chrysovirus 1 RNA3 (CcChv1RNA3)	76%	4E−73	28.37%	hypothetical protein	Diplodia seriata chrysovirus 1 (UOK20163.1)	*Chrysoviridae*	*Alphachrysovirus*	dsRNA
Contig157	PX316568	2,771	Corynespora cassiicola chrysovirus 1 RNA4 (CcChv1RNA4)	90%	0	46.10%	hypothetical protein	Diplodia seriata chrysovirus 1 (UOK20164.1)	*Chrysoviridae*	*Alphachrysovirus*	dsRNA
Contig1249	PX316569	1,820	Corynespora cassiicola partitivirus 1 (CcPav1)	94%	0	82.66%	RdRp	Alternaria tenuissima partitivirus 2 (UCR17162.1)	*Partitiviridae*	*Epsilonpartitivirus*	dsRNA
Contig1246	UIB81488	5,193	Corynespora cassiicola victorivirus 1 (CcVV1)	42%	0	100%	coat protein	Corynespora cassiicola victorivirus 1 (UIB81488.1)	*Totiviridae*	*Victorivirus*	dsRNA
Contig2864	PX316570	2,357	Corynespora cassiicola polymycovirus 1 RNA1 (CcPV1 RNA1)	92%	0	68.51%	RdRp	Plasmopara viticola lesion associated polymycovirus 1 (QHG11067.1)	*Polymycoviridae*	*Polymycovirus*	dsRNA
Contig1012	PX316571	2,240	Corynespora cassiicola polymycovirus 1 RNA2 (CcPV1 RNA2)	95%	0	66.76%	hypothetical protein	Alternaria alternata polymycovirus 1 (QVK45097.1)	*Polymycoviridae*	*Polymycovirus*	dsRNA
Contig635	PX316572	1,494	Corynespora cassiicola polymycovirus 1 RNA3 (CcPV1 RNA3)	93%	0	65.73%	methyl transferase	Plasmopara viticola lesion associated polymycovirus 1 (QHG11068.1)	*Polymycoviridae*	*Polymycovirus*	dsRNA
Contig2009	PX316573	1,112	Corynespora cassiicola polymycovirus 1 RNA4 (CcPV1 RNA4)	71%	2E−126	69.70%	PAS-rich protein	Polymycoviridae sp. (BFW51812.1)	*Polymycoviridae*	*Polymycovirus*	dsRNA

### 3.3 Four novel viruses in the family *Botourmiaviridae*

**Figure 2:**
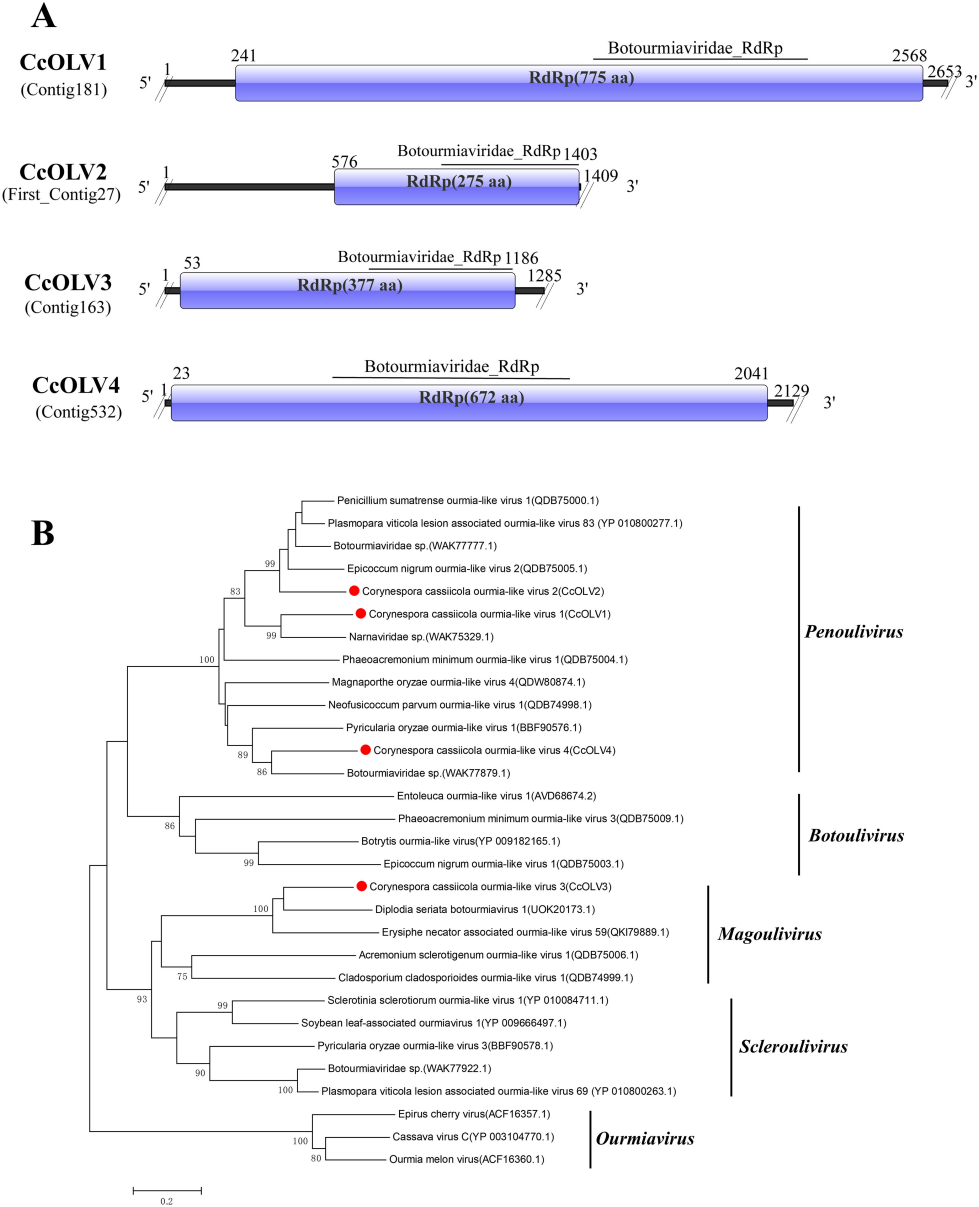
Characterization of the genome structure and phylogenetic analysis of viruses belonging to the *Botourmiaviridae* family. **(A)** Schematic representation of the genome organization of viruses belonging to the *Botourmiaviridae* family. ORFs are indicated by rectangular boxes. Double slashes indicate incomplete genome nucleotide sequences. **(B)** Phylogenetic analysis of the putative *Botourmiaviridae* viruses was performed. The amino acid sequences of the RdRp proteins were aligned using CLUSTAL X, and the phylogenetic tree was constructed with the neighbor-joining (NJ) method. The bootstrap consensus tree (1,000 replicates) is shown, with values ≥ 70% displayed at the branch nodes. Branch lengths are proportional to the number of amino acid substitutions and are measured by a scale bar. CcOLV1, CcOLV2, CcOLV3, and CcOLV4 are indicated in the phylogenetic tree.

Viruses of the family *Botourmiaviridae* infect plants or fungi and have a positive-sense single-stranded RNA genome (Ayllón et al., 2020). In this study, four contig sequences, namely, Contig181 (2,653 bp), First_Contig27 (1,409 bp), Contig163 (1,285 bp), and Contig532 (2,129 bp), showed similarity to viruses of the family *Botourmiaviridae* and all contained the typical domain (Botourmiaviridae_RdRp, cd23183) (**Figure 2A**).

BLASTx alignment using nucleic acid sequences revealed that Contig181 shared 60.03% identity with a virus of the genus *Penoulivirus*, Botourmiaviridae sp. (RdRp, UJQ91941.1), with 78% coverage and an E-value of 0. Contig181 was therefore named Corynespora cassiicola ourmia-like virus 1 (CcOLV1). First_Contig27 showed the highest similarity to Botourmiaviridae sp. (RdRp, WAK77777.1) with 51.3% identity, 97% coverage, and an E-value of 2E−137, and was named Corynespora cassiicola ourmia-like virus 2 (CcOLV2). Contig163 was most similar to Diplodia seriata botourmiavirus 1 (RdRp, UOK20173.1) with 56.65% identity, 87% coverage, and an E-value of 3E−123 (Khan et al., 2022) and was named Corynespora cassiicola ourmia-like virus 3 (CcOLV3). Additionally, Contig532 showed the highest similarity to Botourmiaviridae sp. (RdRp, WAK77879.1) with 57.48% identity, 90% coverage, and an E-value of 0 and was named Corynespora cassiicola ourmia-like virus 4 (CcOLV4) (**Table 1**).

To further clarify the taxonomic status of CcOLV1, CcOLV2, CcOLV3, and CcOLV4, a phylogenetic tree was constructed using the amino acid sequences of the ORF encoding RdRp, along with related viruses from the five genera within the family *Botourmiaviridae*. Phylogenetic tree analysis indicated that CcOLV1, CcOLV2, and CcOLV4 had a closer genetic relationship with viruses of the genus *Penoulivirus*, while CcOLV3 was more closely related to viruses of the genus *Magoulivirus* (**Figure 2B**). Based on the ICTV’s demarcation criteria for viruses of different species within the genera *Penoulivirus* and *Magoulivirus*—namely, that the amino acid sequence identity of the RdRP protein is less than 90%—we therefore hypothesize that the four identified viruses are new species belonging to *the Botourmiaviridae* family.

### 3.4 A related virus in the family *Deltaflexiviridae*

Viruses within the family *Deltaflexiviridae* are linear, positive-sense, single-stranded RNA viruses with molecular masses ranging from 6 to 8 kb. They typically possess four ORFs, encoding the RdRp (ORF1) and three small putative proteins. Their hosts are generally fungi or plant pathogens (Li et al., 2016).

Through sequence alignment, Contig185 (7,931 bp) showed the highest similarity to Sesame deltaflexivirus 1 (SeDV1), specifically to its RdRp (GenBank Accession: QQG34641.1), with 99.57% identity, 76% coverage, and an E-value of 0. This may suggest that Contig185 and SeDV1 are different strains of the same virus. Consequently, Contig185 was named Corynespora cassiicola deltaflexivirus 1 (CcDV1) (**Table 1**). Intriguingly, SeDV1 was isolated from sesame plants, whereas CcDV1 was isolated from the sesame pathogen, *C. cassiicola*. This suggests that during the infection of sesame plants by the *C. cassiicola* strain, a close association involving the bidirectional exchange of cellular contents occurs between the pathogen and the host plant, facilitating viral transmission between the plant and the pathogenic fungus.

**Figure 3:**
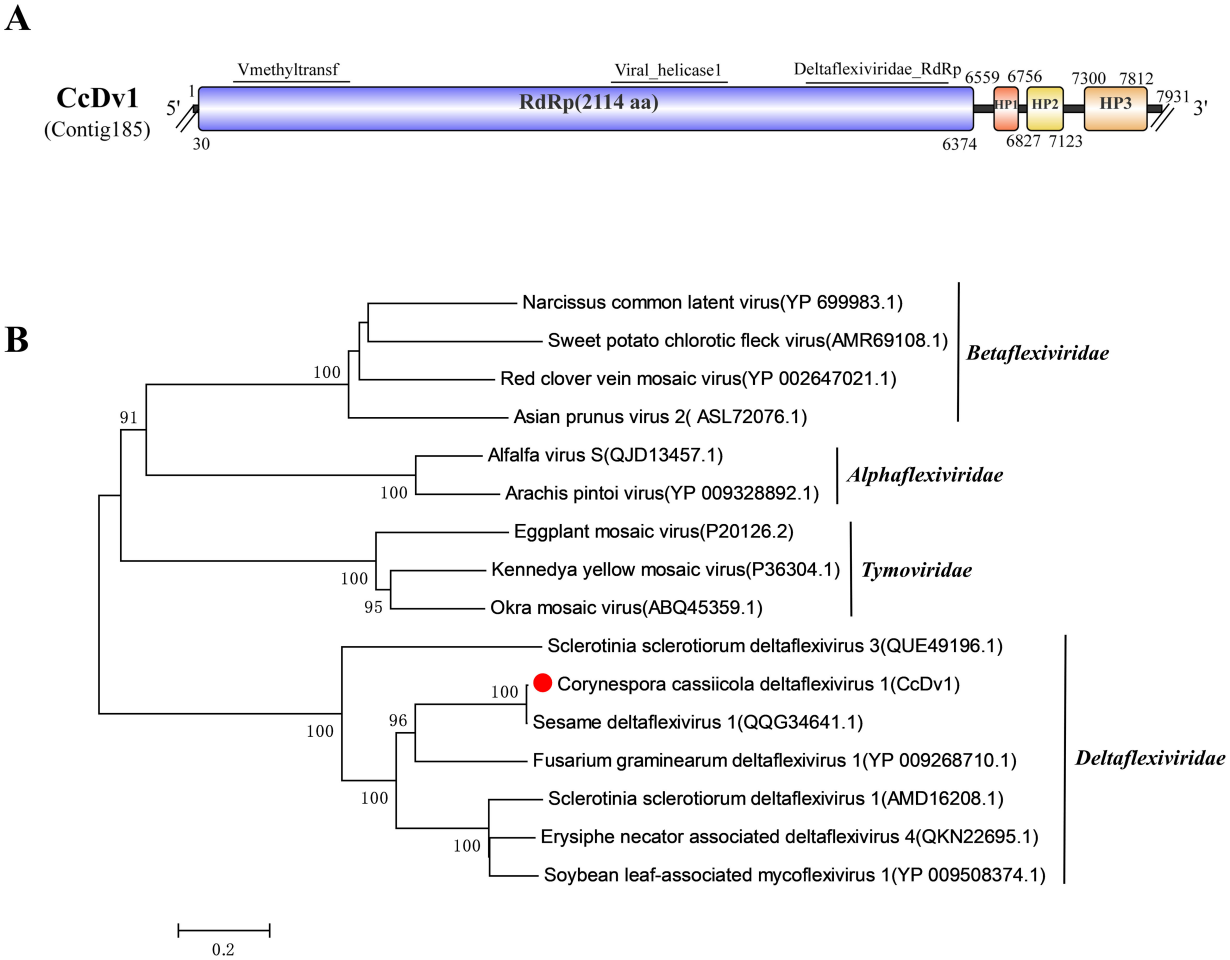
Characterization of the genome structure and phylogenetic analysis of viruses belonging to the *Deltaflexiviridae* family. **(A)** Schematic representation of the genome organization of viruses belonging to the *Deltaflexiviridae* family. ORFs are indicated by rectangular boxes. Double slashes indicate incomplete genome nucleotide sequences. **(B)** Phylogenetic analysis of the putative *Deltaflexiviridae* viruses was performed. The amino acid sequences of the RdRp proteins were aligned using CLUSTAL X, and the phylogenetic tree was constructed with the neighbor-joining (NJ) method. The bootstrap consensus tree (1,000 replicates) is shown, with values ≥ 70% displayed at the branch nodes. Branch lengths are proportional to the number of amino acid substitutions and are measured by a scale bar. CcDv1 is indicated in the phylogenetic tree.

Comparative analysis of the predicted gene structures revealed that CcDV1 and SeDV1 both possess four ORFs. ORF1 contains three conserved domains: Vmethyltransf (pfam01660), Viral_helicase1 (pfam01443), and Deltaflexiviridae_RdRp (cd23248). The other three ORFs encode putative proteins of unknown function. This domain architecture is characteristic of viruses within the family *Deltaflexiviridae* (**Figure 3A**).

A phylogenetic tree was constructed based on the amino acid sequence of the ORF1 (RdRp) of CcDV1 and representative viruses from the four families within the order *Tymovirales*: *Deltaflexiviridae*, *Tymoviridae*, *Alphaflexiviridae*, and *Betaflexiviridae*. The tree shows that CcDV1 and SeDV1 cluster closely with related viruses belonging to the family *Deltaflexiviridae* (**Figure 3B**). This represents the first report of a virus belonging to the family *Deltaflexiviridae* identified in the sesame pathogen *C. cassiicola*.

### 3.5 Two related virus in the family *Fusariviridae*

Viruses within the family *Fusariviridae* are positive-sense, single-stranded RNA viruses with genome sizes ranging from 5.9 to 10.7 kb. They can contain up to four predicted ORFs, encoding the RdRp (ORF1) and three small putative proteins. Their hosts are primarily confirmed to be organisms within the fungal kingdom (Chiba et al., 2024).

**Figure 4:**
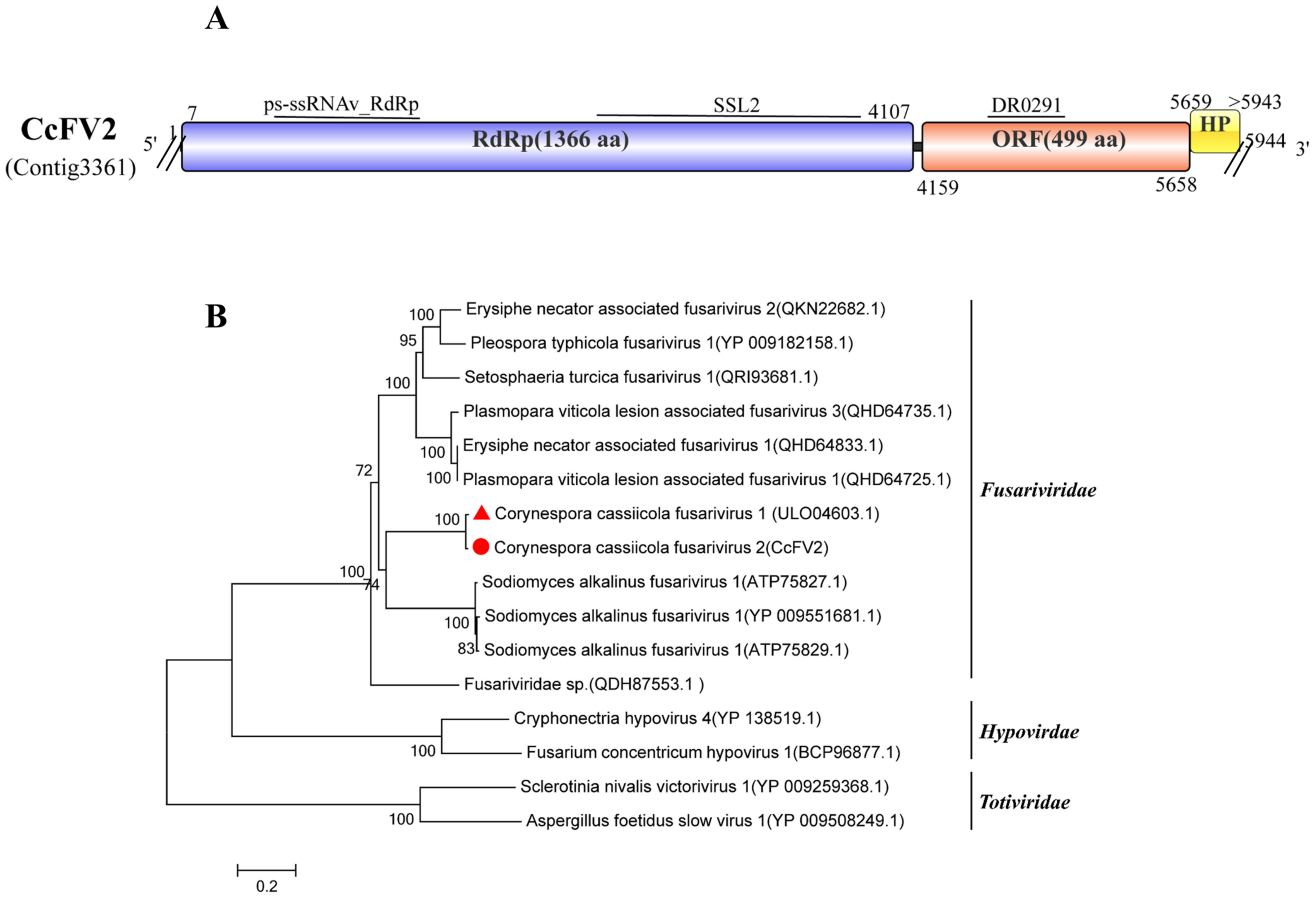
Characterization of the genome structure and phylogenetic analysis of viruses belonging to the *Fusariviridae* family. **(A)** Schematic representation of the genome organization of viruses belonging to the *Fusariviridae* family. ORFs are indicated by the rectangular boxes. Double slashes indicate incomplete genome nucleotide sequences. **(B)** Phylogenetic analysis of the putative *Fusariviridae* viruses was performed. The amino acid sequences of the RdRp proteins were aligned using CLUSTAL X, and the phylogenetic tree was constructed with the neighbor-joining (NJ) method. The bootstrap consensus tree (1,000 replicates) is shown, with values ≥ 70% displayed at the branch nodes. Branch lengths are proportional to the number of amino acid substitutions and are measured by a scale bar. CcFV1 and CcFV2 are indicated in the phylogenetic tree.

Sequence alignment revealed that Contig3361 (5,944 bp) showed the highest similarity to Corynespora cassiicola fusarivirus 1 (CcFV1), which is a virus we reported previously, specifically to its RdRp (GenBank Accession: ULO04603.1), with 69% coverage, an E-value of 0, and 98.32% identity (Liu et al., 2022a). This suggests that Contig3361 and CcFV1(Contig2308) are different strains of the same virus. Therefore, Contig3361 was named Corynespora cassiicola fusarivirus 2 (CcFV2). CcFV2 possesses three ORFs. ORF1 contains two conserved domains: ps-ssRNAv-RdRp (cd23170) and SSL2 (COG1061). ORF2 possesses the DR0291-conserved domain (cl34310) (**Figure 4A**).

To investigate the relationship between CcFV2 and other fungal viruses, a phylogenetic tree was constructed based on the polypeptide encoded by ORF1, which contains the RdRp domain. The results showed that based on the RdRp, CcFV2 and CcFV1 clustered together with viruses belonging to the family *Fusariviridae*. They formed a distinct clade separate from members of the family *Hypoviridae* and *Totiviridae* (**Figure 4B**). Based on analysis of the gene structure and the phylogenetic tree, CcFV2 is considered a member of the family *Fusariviridae*.

### 3.6 Two novel virus in the family *Narnaviridae*

Members of the family *Narnaviridae* possess genomes ranging from 2.5 to 2.9 kb. They are unencapsidated, positive-sense, single-stranded RNA viruses that encode only a single protein, which is the simplest of RNA viruses (Hillman and Cai, 2013).

**Figure 5:**
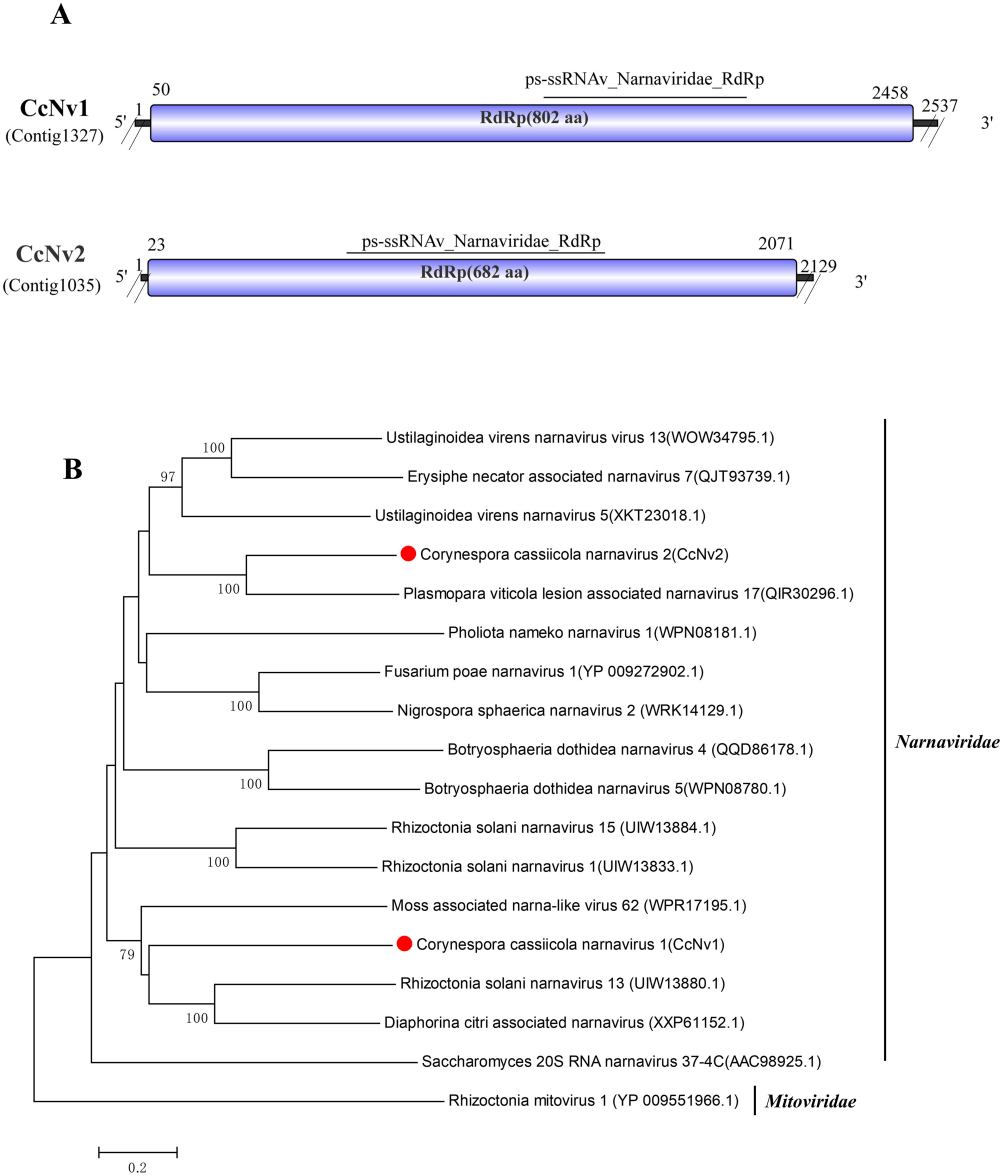
Characterization of the genome structure and phylogenetic analysis of viruses belonging to the *Narnaviridae* family. **(A)** Schematic representation of the genome organization of viruses belonging to the *Narnaviridae* family. ORFs are indicated by the rectangular boxes. Double slashes indicate incomplete genome nucleotide sequences. **(B)** Phylogenetic analysis was performed on viruses belonging to the families *Narnaviridae* and *Mitoviridae*. The amino acid sequences of the RdRp proteins were aligned using CLUSTAL X, and the phylogenetic tree was constructed with the neighbor-joining (NJ) method. The bootstrap consensus tree (1,000 replicates) is shown, with values ≥ 70% displayed at the branch nodes. Branch lengths are proportional to the number of amino acid substitutions and are measured by a scale bar. CcNv1 and CcNv2 are indicated in the phylogenetic tree.

Sequence alignment analysis identified Contig1327 (2,537 bp) and Contig1035 (2,129 bp) as belonging to the family *Narnaviridae*. Contig1327 showed the highest similarity to Rhizoctonia solani narnavirus 13 based on its RdRp (GenBank Accession: UIW13880.1), with 33.14% identity, 57% coverage, and an E-value of 1E−55 (Abdoulaye et al., 2019). We named this contig Corynespora cassiicola narnavirus 1 (CcNv1). Contig1035 showed the highest similarity to Plasmopara viticola lesion associated narnavirus 17 based on its RdRp (GenBank Accession: QIR30296.1), with 38.91% identity, 96% coverage, and an E-value of 2E−137 (Chiapello et al., 2020). We named this contig Corynespora cassiicola narnavirus 2 (CcNv2). Conserved domain prediction revealed that both CcNv1 and CcNv2 possess the ps-ssRNAv_Narnaviridae_RdRp (cd23177) domain (**Figure 5A**).

To further clarify the taxonomic position of these two viruses, a phylogenetic tree was constructed using the amino acid sequences of their RdRp-encoding ORFs alongside representative viruses from the *Narnaviridae* and *Mitoviridae*. The results indicated that CcNv1 and CcNv2 grouped within the same clade as viruses belonging to the family *Narnaviridae*. According to the ICTV criteria for the genus *Narnavirus*, an amino acid sequence identity of less than 50% typically demarcates a new species. The RdRp of CcNv1 and CcNv2 shares only 33.14% and 38.91% identity, respectively, with their closest relatives among known narnaviruses. Therefore, CcNv1 and CcNv2 represent two novel species within the genus *Narnavirus*. Furthermore, this study reports the first identification of narnaviruses in *C. cassiicola*. So, these two viruses are proposed to be new members of the genus *Narnavirus* within the family *Narnaviridae* (**Figure 5B**).

### 3.7 Two related virus in the family Ambiguiviridae

**Figure 6:**
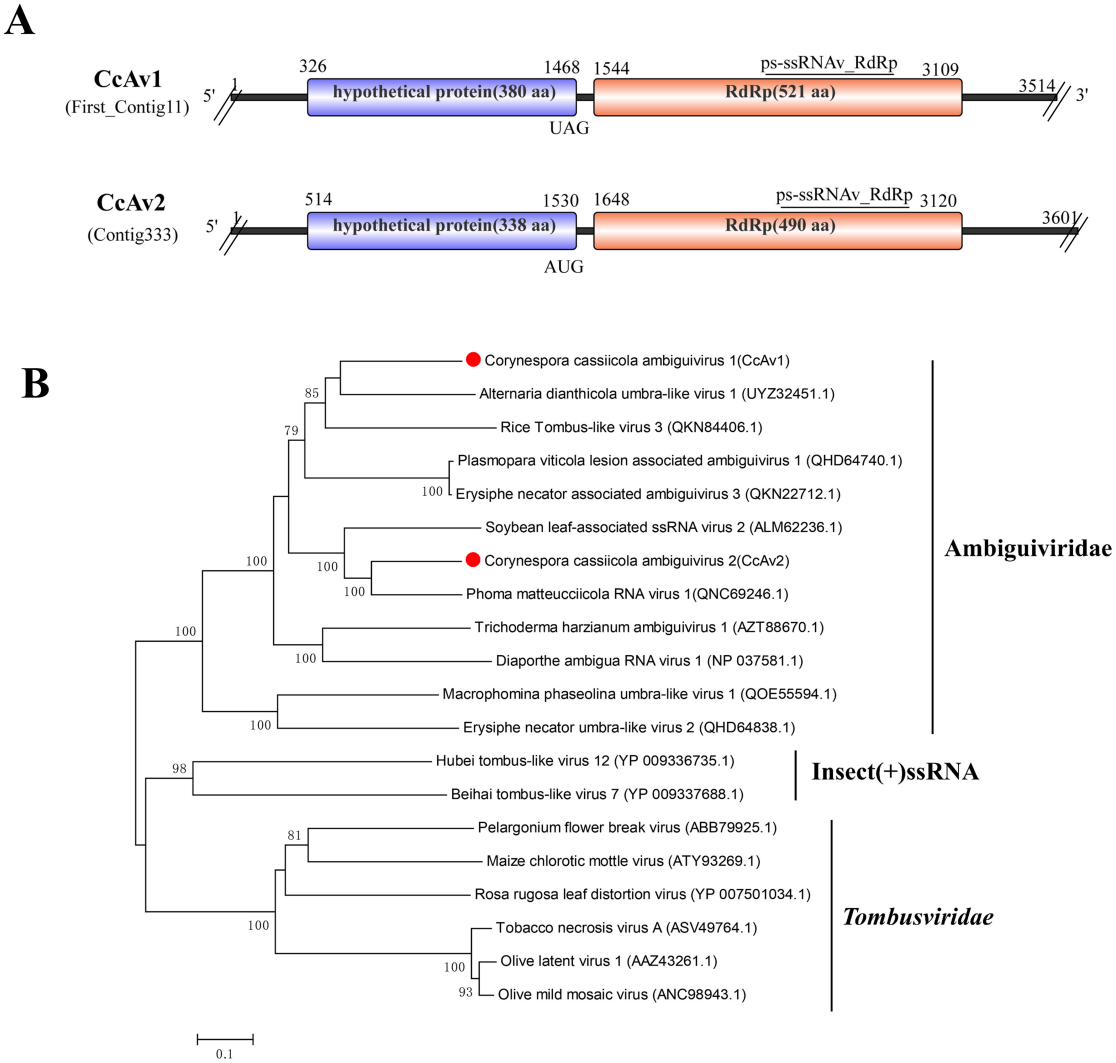
Characterization of the genome structure and phylogenetic analysis of viruses belonging to the Ambiguiviridae family. **(A)** Schematic representation of CcAv1 and CcAV2. The genomes of CcAv1 and CcAV2 possessed two ORFs, encoding a hypothetical protein and an RdRP, respectively. Double slashes indicate incomplete genome nucleotide sequences. **(B)** Phylogenetic analysis of the putative Ambiguiviridae viruses was performed. The amino acid sequences of the RdRp proteins were aligned using CLUSTAL X, and the phylogenetic tree was constructed with the neighbor-joining (NJ) method. The bootstrap consensus tree (1,000 replicates) is shown, with values ≥ 70% displayed at the branch nodes. Branch lengths are proportional to the number of amino acid substitutions and are measured by a scale bar. CcAv1 and CcAv2 are indicated in the phylogenetic tree.

Two contigs showed similarity to viruses within the newly proposed family Ambiguiviridae (Gilbert et al., 2019). These viruses, First_Contig11 (3,514 bp) and Contig333 (3,601 bp), each contain two ORFs, with the second ORF encoding the RdRp. They were named CcAv1 and CcAv2, respectively (**Figure 6A**).

BLASTx searches revealed that CcAv1 and CcAv2 share high similarity with RdRps of several unclassified viruses: CcAv1 showed its closest match to Alternaria dianthicola umbra-like virus 1 (GenBank Accession: UYZ32451.1), with 61.45% identity, 42% coverage, and an E-value of 0 (Zhong et al., 2022). CcAv2 showed its closest match to Phoma matteucciicola RNA virus 1 (GenBank Accession: QNC69246.1), with 71.52% identity, 40% coverage, and an E-value of 0 (Zhou et al., 2021a). Traditionally, most (+)ssRNA viruses possess the GDD sequence within the catalytic motif of their RdRp. However, these two fungal viruses, along with the selected Ambiguiviridae members, possess an uncommon GDN sequence in their RdRp motif (**Supplementary Figure 4**). This specific sequence feature aligns with those previously identified for members of the proposed family Ambiguiviridae (Gilbert et al., 2019; Zhong et al., 2022; Buivydaitė et al., 2024).

Phylogenetic analysis based on the RdRp domains of CcAv1 and CcAv2, alongside other selected viruses, showed that CcAv1 and CcAv2 clustered together with other reported fungal RNA viruses (Ambiguiviridae). This cluster formed a distinct clade, clearly separated from related viruses in the family *Tombusviridae* and unclassified insect-associated viruses (**Figure 6B**). Although the taxonomic classification of members within the newly proposed family “Ambiguiviridae” has not yet been formally established, based on their high sequence similarity and shared characteristics, CcAv1 and CcAv2 should be classified as members within this proposed family.

### 3.8 A related virus in the family *Potyviridae*

*Potyviridae* represents the largest family of plant RNA viruses, accounting for approximately 30% of known plant viruses. It comprises 12 genera, with *Potyvirus* being the largest genus within the family. Virions consist of flexuous filamentous particles with helical symmetry and contain infectious, linear, positive-sense, single-stranded RNA genomes (Inoue-Nagata et al., 2022).

**Figure 7:**
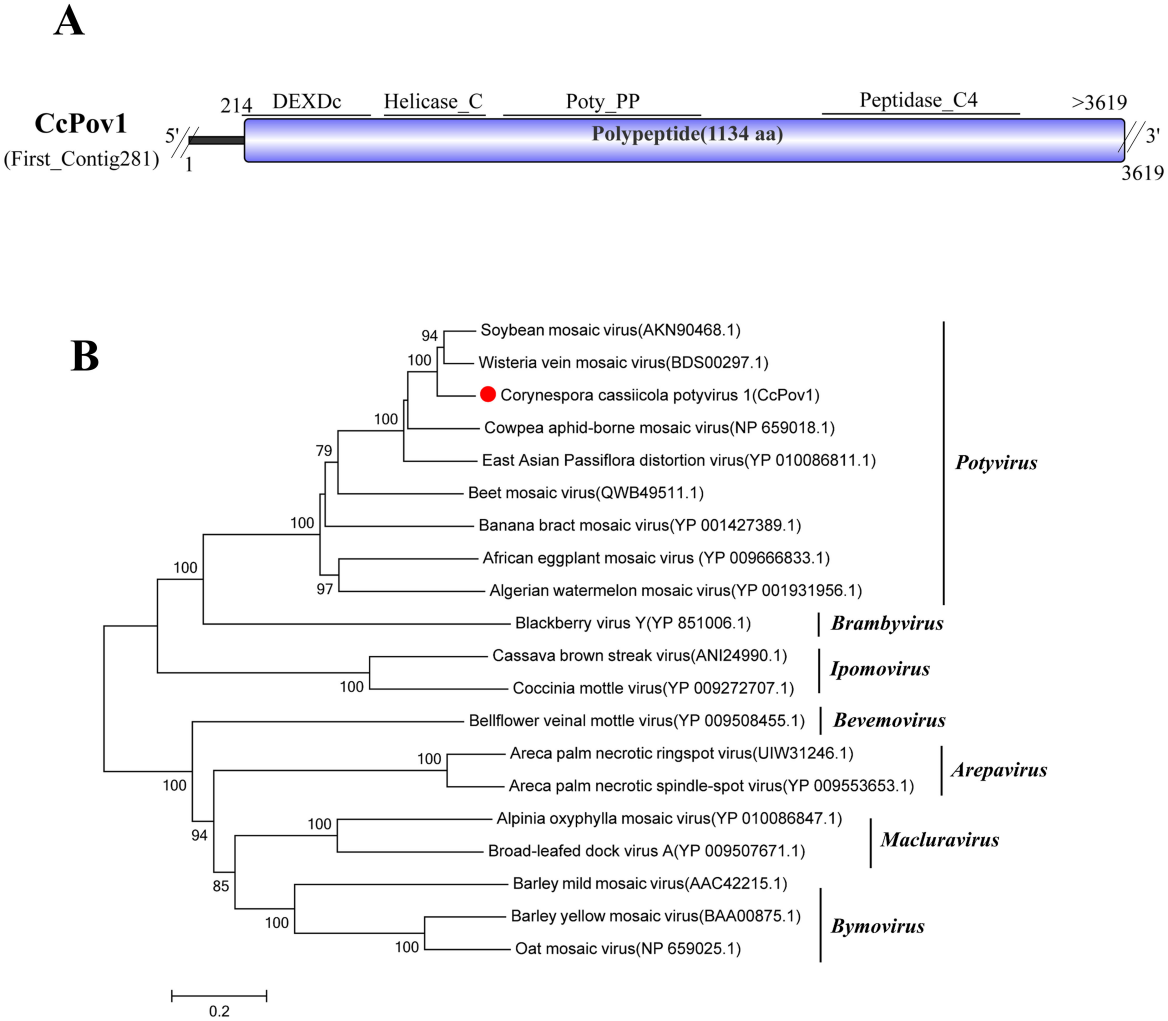
Characterization of the genome structure and phylogenetic analysis of viruses belonging to the *Potyviridae* family. **(A)** Schematic representation of the genome organization of viruses belonging to the *Potyviridae* family. ORF is indicated by a rectangular box. Double slashes indicate incomplete genome nucleotide sequences. **(B)** Phylogenetic analysis of the putative *Potyviridae* viruses was performed. The amino acid sequences of the RdRp proteins were aligned using CLUSTAL X, and the phylogenetic tree was constructed with the neighbor-joining (NJ) method. The bootstrap consensus tree (1,000 replicates) is shown, with values ≥ 70% displayed at the branch nodes. Branch lengths are proportional to the number of amino acid substitutions and are measured by a scale bar. CcPov1 is indicated in the phylogenetic tree.

Sequence analysis revealed that First_Contig281 (3,619 bp) shares high similarity with Wisteria vein mosaic virus (WvMV), specifically its polyprotein (GenBank Accession: BDS00297.1), exhibiting 84.25% identity, 100% coverage, and an E-value of 0 (Yin et al., 2023). Consequently, this contig was named Corynespora cassiicola potyvirus 1 (CcPov1). Analysis of its conserved domains showed that the ORF encodes multiple domains: DEXDc (SMART00487), Helicase_C (PF00271), Poly_PP (CL07169), and Peptidase_C4 (CL24133). However, compared with the full-length WvMV genome, CcPov1 lacks the Peptidase_S30 (CL44322) and Peptidase_C6 (CL20022) domains at its 5′ terminus and the Potyviridae_RdRp (CD23175) and Poty_coat (CL02961) domains at its 3′ terminus (**Figure 7A**).

Phylogenetic analysis based on the amino acid sequence of the polypeptide encoded by the CcPov1 ORF demonstrated that CcPov1 clusters within a branch containing members of the genus *Potyvirus* in the family *Potyviridae*, suggesting that CcPov1 is a member of the genus *Potyvirus* (**Figure 7B**).

### 3.9 One novel virus and one related virus in the family *Mymonaviridae*

As reported by Jiāng et al (Jiāng et al., 2022), viruses within the family *Mymonaviridae* infect fungal hosts. They possess enveloped, filamentous virions with diameters ranging from 25 to 50 nm. Their genomes are linear, negative-sense, single-stranded RNA molecules.

BLASTx analysis of two viral sequences, Contig27 (6,519 bp) and Contig28 (6,619 bp), revealed that both share the highest similarity with the RdRp domain of grapevine wood holobiome associated mononegaambi virus 2 (GWHaMOV2) (GenBank Accession: XLV09868.1). The identities were 70.25% and 67.08%, respectively, both with 88% coverage and an E-value of 0 (Debat et al., 2025). We named Contig27 Corynespora cassiicola mymonavirus 1 (CcMV1) and Contig28 Corynespora cassiicola mymonavirus 2 (CcMV2).

**Figure 8:**
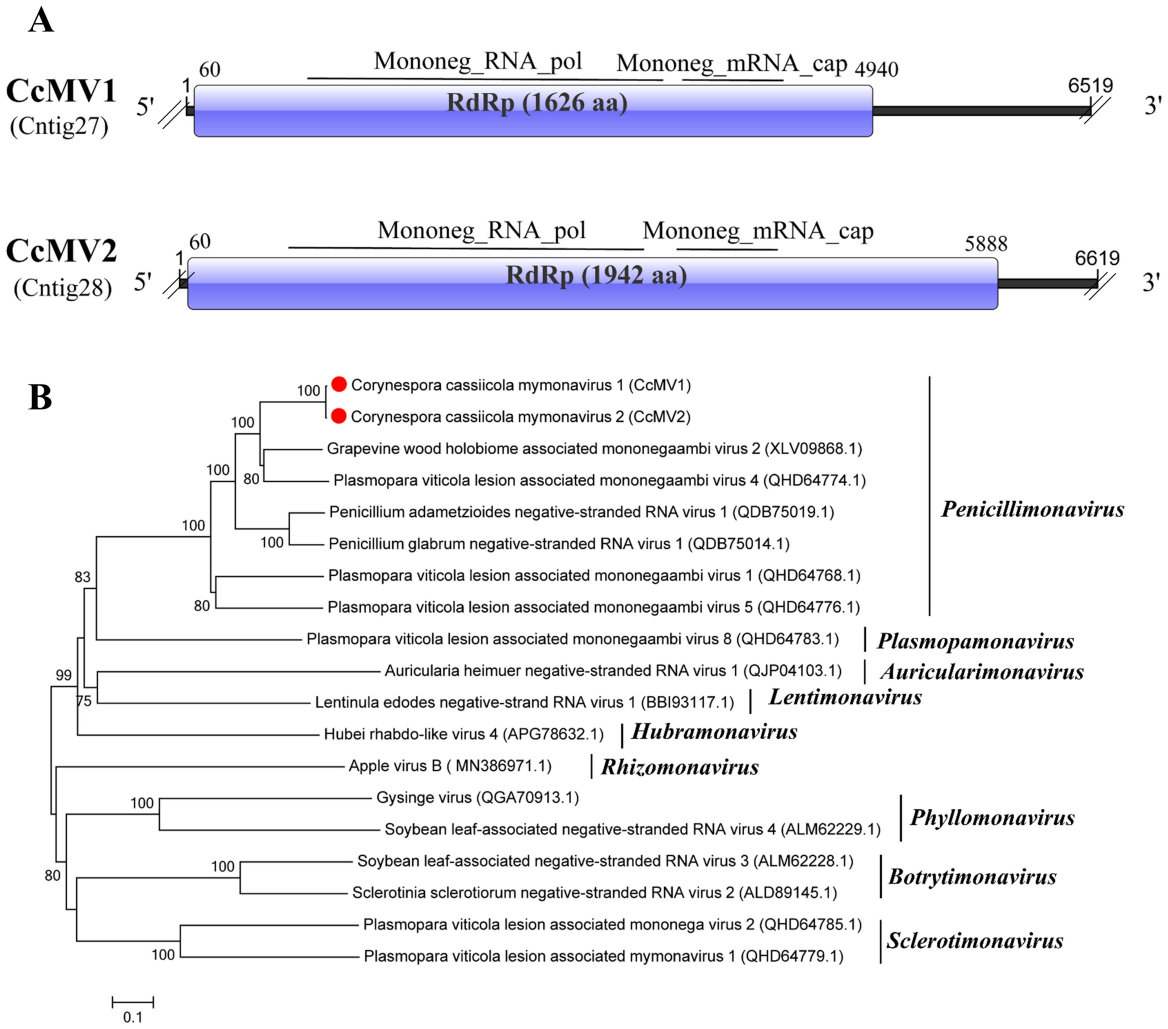
Characterization of the genome structure and phylogenetic analysis of viruses belonging to the *Mymonaviridae* family. **(A)** Schematic representation of the genome organization of viruses belonging to the *Mymonaviridae* family. ORFs are indicated by the rectangular boxes. Double slashes indicate incomplete genome nucleotide sequences. **(B)** Phylogenetic analysis of the putative *Mymonaviridae* viruses was performed. The amino acid sequences of the RdRp proteins were aligned using CLUSTAL X, and the phylogenetic tree was constructed with the neighbor-joining (NJ) method. The bootstrap consensus tree (1,000 replicates) is shown, with values ≥ 70% displayed at the branch nodes. Branch lengths are proportional to the number of amino acid substitutions and are measured by a scale bar. CcMV1 and CcMV2 are indicated in the phylogenetic tree.

Gene structure schematic analysis showed that CcMV1 and CcMV2 possess two conserved domains: Mononeg_RNA_pol (pfam00946) and Mononeg_mRNAcap (pfam14318). The three viruses share similar nucleic acid lengths and genomic organizations (**Figure 8A**). To further elucidate the phylogenetic relationship of CcMV1 and CcMV2, a neighbor-joining phylogenetic tree was constructed using the amino acid sequences of the RdRp-encoding ORF from representative viruses across the nine genera within the family *Mymonaviridae*. The results showed that CcMV1 and CcMV2 clustered within a clade containing viruses of the genus *Penicillimonavirus* (**Figure 8B**).

Based on predicted gene structures and phylogenetic analysis, both CcMV1 and CcMV2 show similarity to viruses in the genus *Penicillimonavirus* (family *Mymonaviridae*). According to ICTV species demarcation criteria for this genus, distinct species must exhibit >30% divergence in both nucleoprotein amino acid sequence and coding-complete genome nucleotide sequence. Sequence comparison revealed that CcMV2 shows less than 70% nucleotide sequence identity (i.e., over 30% divergence) to all known viruses in this genus, meeting the criterion for new species designation. Therefore, we propose that while CcMV1 belongs to an established species in *Penicillimonavirus*, CcMV2 represents a novel species within this genus.

### 3.10 A related virus in the family Mycophioviridae

The family Mycophioviridae was newly proposed by Nerva et al. in 2019 (Nerva et al., 2019). During phylogenetic analysis of Cladosporium cladosporioides negative-stranded RNA virus 1 (CcRV1), this virus showed distinct separation from members of the genus *Ophiovirus* within the family *Aspiviridae*. Based on this finding, viruses clustering with CcRV1 were reclassified into a novel family designated Mycophioviridae (Nerva et al., 2019; Chiapello et al., 2020). The proposed virus family is merely a recently suggested hypothesis and has not yet been formally submitted to the ICTV as an official proposal.

Sequence analysis of Contig47 (7,265 bp) revealed high similarity to grapevine wood holobiome-associated mycophiovirus 1 (GenBank Accession: XNS37001.1) (Debat et al., 2025), with 76.44% identity, 97% coverage, and an E-value of 0. We named Contig47 Corynespora cassiicola negative-stranded RNA virus 1 (CcRV1).

**Figure 9:**
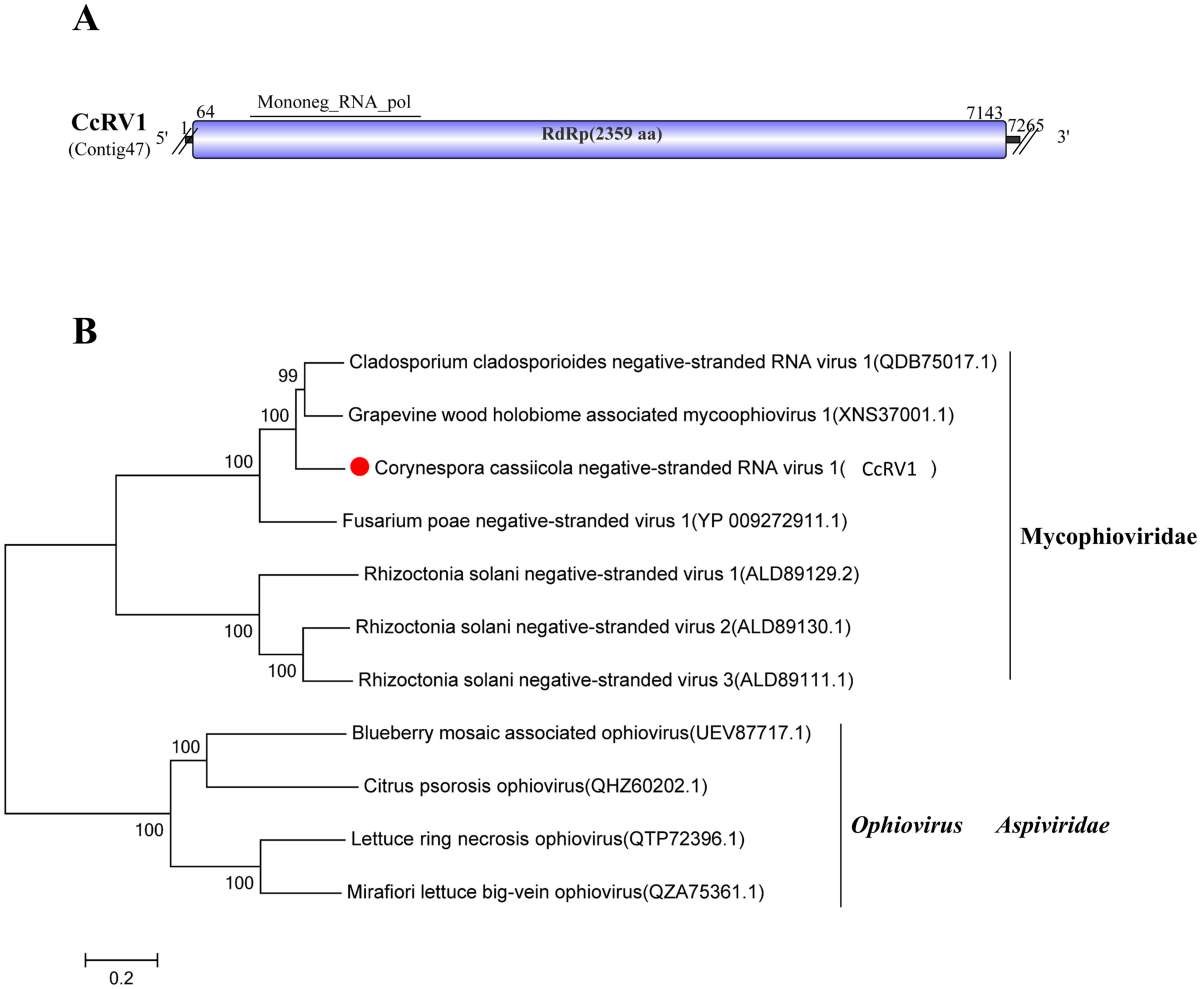
Characterization of the genome structure and phylogenetic analysis of viruses belonging to the Mycophioviridae family. **(A)** Schematic representation of the genome organization of viruses belonging to the Mycophioviridae family. ORF is indicated by a rectangular box. Double slashes indicate incomplete genome nucleotide sequences. **(B)** Phylogenetic analysis of the putative Mycophioviridae viruses was performed. The amino acid sequences of the RdRp proteins were aligned using CLUSTAL X, and the phylogenetic tree was constructed with the neighbor-joining (NJ) method. The bootstrap consensus tree (1,000 replicates) is shown, with values ≥ 70% displayed at the branch nodes. Branch lengths are proportional to the number of amino acid substitutions and are measured by a scale bar. CcRV1 is indicated in the phylogenetic tree.

Comparative analysis of its genomic structure showed that CcRV1 possesses the conserved Mononeg_RNA_pol (pfam00946) domain (**Figure 9A**). Phylogenetic analysis based on the amino acid sequence of the viral protein further confirmed that CcRV1 clusters with established members of the family Mycophioviridae (**Figure 9B**). Thus, CcRV1 is proposed to be a member of the newly proposed family Mycophioviridae.

### 3.11 A novel virus in the family *Chrysoviridae*

Viruses within the family *Chrysoviridae* typically possess four genomic segments of dsRNA, each packaged within separate particles. These segments form a genome size ranging from 8.9 to 16 kbp. Viral replication often occurs in cytoplasmic aggregates. Chrysoviruses infect fungi, plants, and insects and may cause hypovirulence (attenuated pathogenicity) in their fungal hosts (Kotta-Loizou et al., 2020).

**Figure 10:**
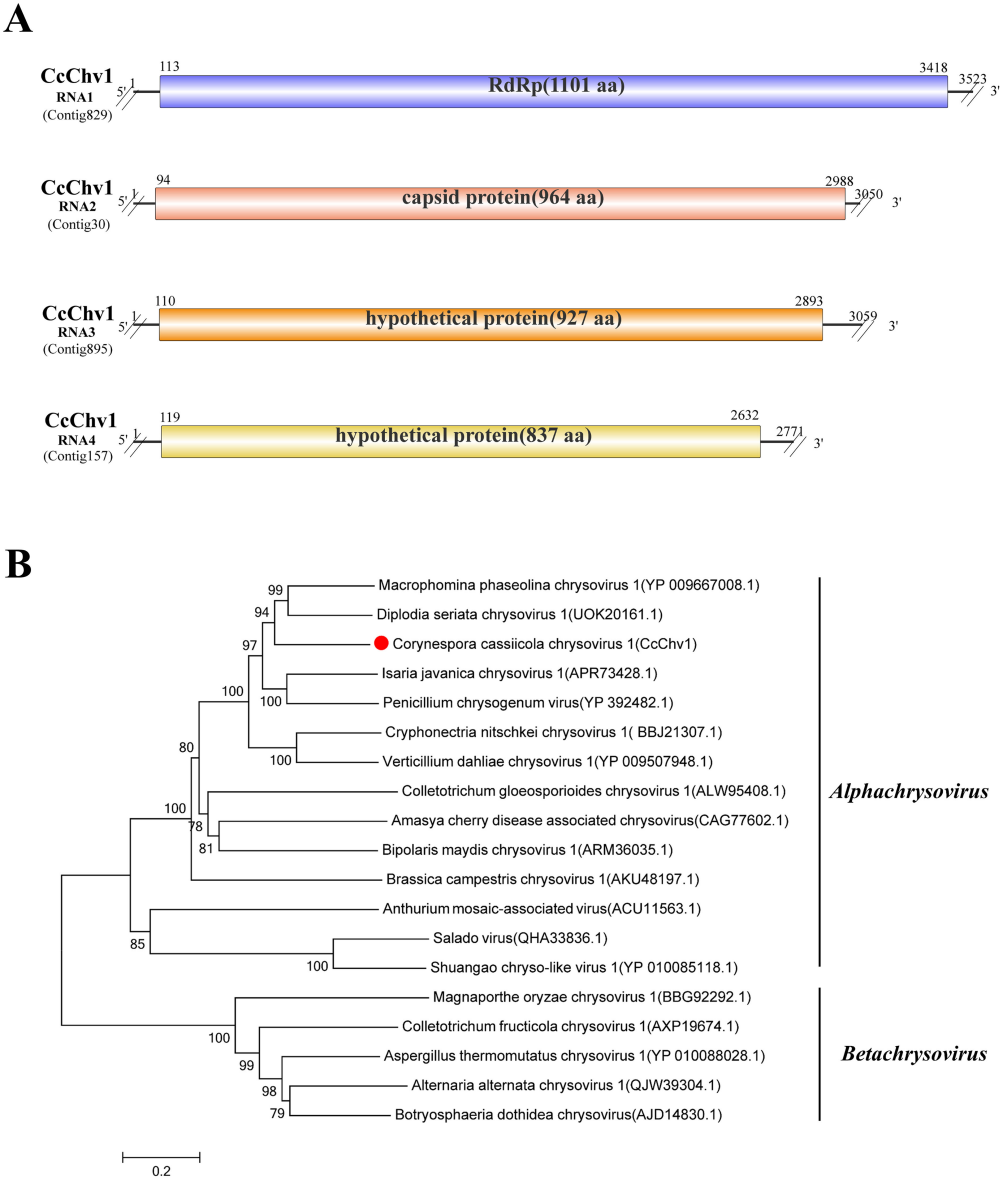
Characterization of the genome structure and phylogenetic analysis of viruses belonging to the *Chrysoviridae* family. **(A)** Schematic representation of the genome organization of viruses belonging to the *Chrysoviridae* family. ORFs are indicated by rectangular boxes. Double slashes indicate incomplete genome nucleotide sequences. **(B)** A phylogenetic analysis of the putative *Chrysoviridae* viruses was performed. The amino acid sequences of the RdRp proteins were aligned using CLUSTAL X, and the phylogenetic tree was constructed with the neighbor-joining (NJ) method. The bootstrap consensus tree (1,000 replicates) is shown, with values ≥ 70% displayed at the branch nodes. Branch lengths are proportional to the number of amino acid substitutions and are measured by a scale bar. CcChv1 is indicated in the phylogenetic tree.

Sequence alignment analysis of Contig829 (3,523 bp), Contig30 (3,050 bp), Contig895 (3,059 bp), and Contig157 (2,771 bp) revealed that each contig showed the highest similarity to different proteins encoded by Diplodia seriata chrysovirus 1 (DsCV1) (Khan et al., 2022): Contig829 matched the RdRp (UOK20161.1; 59.03% identity, 94% coverage, E-value 0); Contig30 matched the capsid protein (CP) (UOK20162.1; 35.35% identity, 94% coverage, E-value 0); Contig895 matched a hypothetical protein (UOK20163.1; 28.37% identity, 76% coverage, E-value 4E−73); and Contig157 matched a hypothetical protein (UOK20164.1; 46.1% identity, 90% coverage, E-value 0) (**Figure 10A**; **Table 1**). RT-PCR analysis confirmed that all four contigs were exclusively detected in strain N3 (**Supplementary Figure 2**). This may suggest that these contigs represent the four segments of a single chrysovirus, putatively based on their co-occurrence and homology. Consequently, the virus was named Corynespora cassiicola chrysovirus 1 (CcChv1). To clarify the relationship of CcChv1 to the family *Chrysoviridae*, a phylogenetic tree was constructed using the amino acid sequences of the RdRp from CcChv1 and representative viruses of the genera *Alphachrysovirus* and *Betachrysovirus*. The results showed that CcChv1 clustered within a clade containing viruses of the genus *Alphachrysovirus* (**Figure 10B**).

CcChv1 is proposed as a novel member of the family *Chrysoviridae* based on its distinct phylogenetic position, a four-segmented genome encoding an RdRp, a CP, and hypothetical proteins, and RdRp/CP sequence identities below the species demarcation thresholds (≤70% and ≤53%, respectively). This represents the first report of an *Alphachrysovirus* infecting *C. cassiicola.*

### 3.12 A related virus in the family *Partitiviridae*

Viruses within the family *Partitiviridae* infect plants, fungi, and protozoans. They possess two double-stranded RNA genome segments, with total genome sizes typically ranging from 3.0 to 4.8 kbp. Segment dsRNA1 encodes the RdRp, and segment dsRNA2 encodes the capsid protein (CP) (Vainio et al., 2018). The family has five genera, with characteristic hosts for members of each genus. Moreover, recently, two novel genera, *Epsilonpartitivirus* and *Zetapartitivirus*, have been proposed (Nerva et al., 2017; Jiang et al., 2019; Taggart et al., 2023).

**Figure 11:**
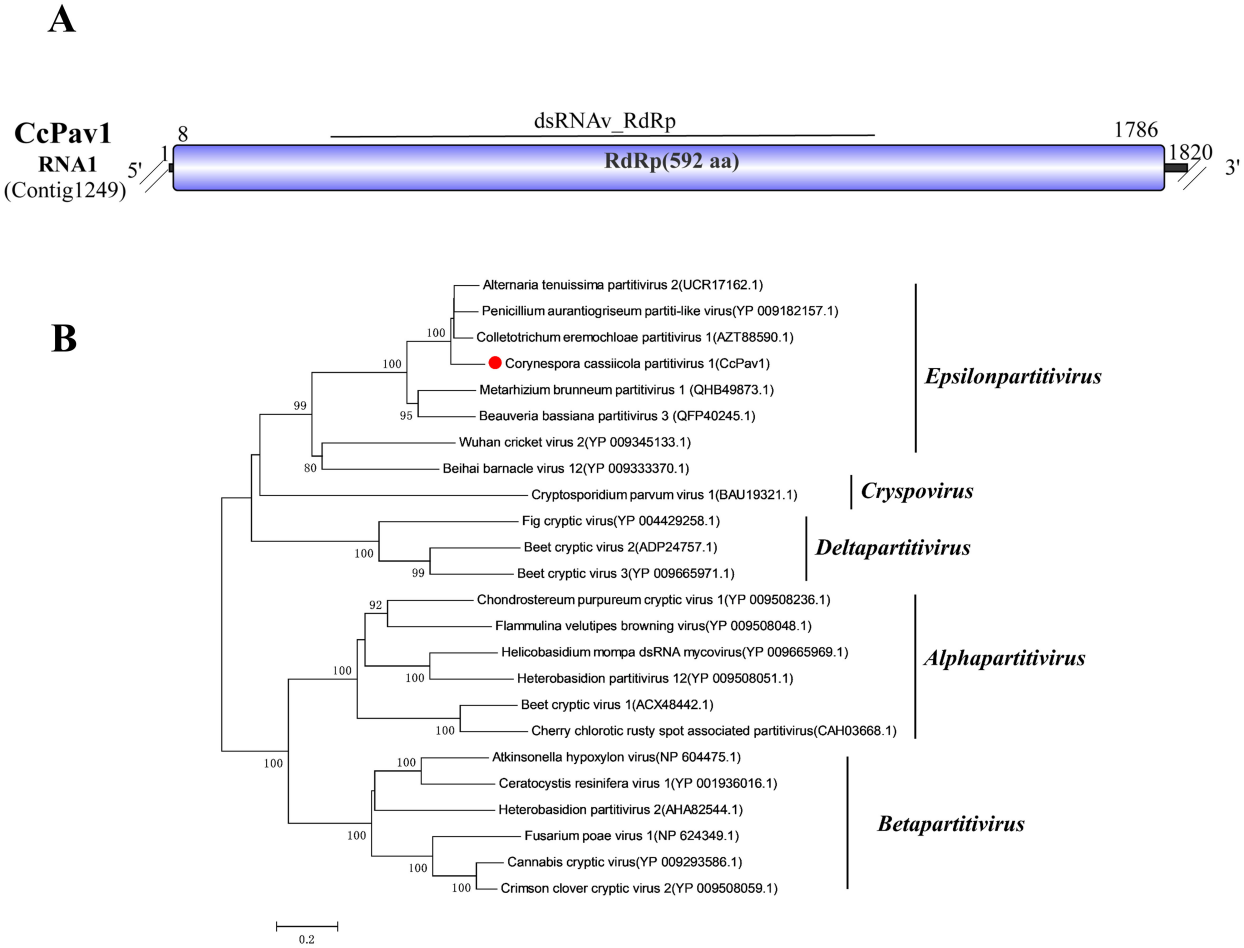
Characterization of the genome structure and phylogenetic analysis of viruses belonging to the *Partitiviridae* family. **(A)** Schematic representation of the genome organization of viruses belonging to the *Partitiviridae* family. ORF is indicated by a rectangular box. Double slashes indicate incomplete genome nucleotide sequences. **(B)** A phylogenetic analysis of the putative *Partitiviridae* viruses was performed. The amino acid sequences of the RdRp proteins were aligned using CLUSTAL X, and the phylogenetic tree was constructed with the neighbor-joining (NJ) method. The bootstrap consensus tree (1,000 replicates) is shown, with values ≥ 70% displayed at the branch nodes. Branch lengths are proportional to the number of amino acid substitutions and are measured by a scale bar. CcPav1 is indicated in the phylogenetic tree.

Sequence alignment of Contig1249 (1,820 bp) revealed high similarity to the RdRp of Alternaria tenuissima partitivirus 2 (UCR17162.1; 82.66% identity, 94% coverage, E-value 0) (Wang et al., 2022). Consequently, this contig was named Corynespora cassiicola partitivirus 1 (CcPav1). Conserved domain prediction identified the dsRNAv_RdRp (cd23185) domain in CcPav1. However, we did not detect the dsRNA2 segment encoding the CP (CcPav1 RNA2) in our sequencing results (**Figure 11A**).

To further elucidate the phylogenetic position of CcPav1, a phylogenetic tree was constructed using the amino acid sequence of the ORF encoded by CcPav1 RNA1 alongside representative viruses from the established and proposed genera within the family *Partitiviridae* (**Figure 11B**). The results indicated that CcPav1 is closely related to members of the newly proposed genus *Epsilonpartitivirus*.

Thus, based on the genomic structure and the phylogenetic analysis, CcPav1 is a member of the genus *Epsilonpartitivirus*.

### 3.13 A novel virus in the family *Polymycoviridae*

Viruses within the family *Polymycoviridae* possess multisegmented, unconventionally encapsidated double-stranded RNA genomes. Typically comprising four genomic segments (although some contain up to eight), their complete genomes range from 7.5 to 12.5 kbp. These viruses infect fungi or oomycetes and can alter host growth and virulence (Kotta-Loizou et al., 2022).

Sequence alignment of Contig2864 (2,357 bp), Contig1012 (2,240 bp), Contig635 (1,494 bp), and Contig2009 (1,112 bp) revealed that each contig corresponds to distinct segments of polymycoviruses: Contig2864 matches Plasmopara viticola lesion-associated polymycovirus 1 (PvlaPv1, RdRp, QHG11067.1, 68.51% identity, 92% coverage, E-value 0) (Chiapello et al., 2020); Contig1012 matches Alternaria alternata polymycovirus 1 (AaPV1, hypothetical protein, QVK45097.1, 66.76% identity, 95% coverage, E-value 0) (Ma et al., 2022); Contig635 matches the methyltransferase of PvlaPv1 (QHG11068.1, 65.73% identity, 93% coverage, Evalue 0) (Chiapello et al., 2020); and Contig2009 matches Polymycoviridae sp. (AaPV1, hypothetical protein, BFW51812.1, 69.7% identity, 71% coverage, E-value 2E-126) (Samir et al., 2025). All four segments were assigned to the *Polymycoviridae* family. In addition, RT-PCR confirmed their exclusive presence in strain N2, indicating that they constitute segments of a single virus. This virus was designated Corynespora cassiicola polymycovirus 1 (CcPV1), with the contigs named CcPV1 RNA1 (Contig2864), CcPV1 RNA2 (Contig1012), CcPV1 RNA3 (Contig635), and CcPV1 RNA4 (Contig2009).

**Figure 12:**
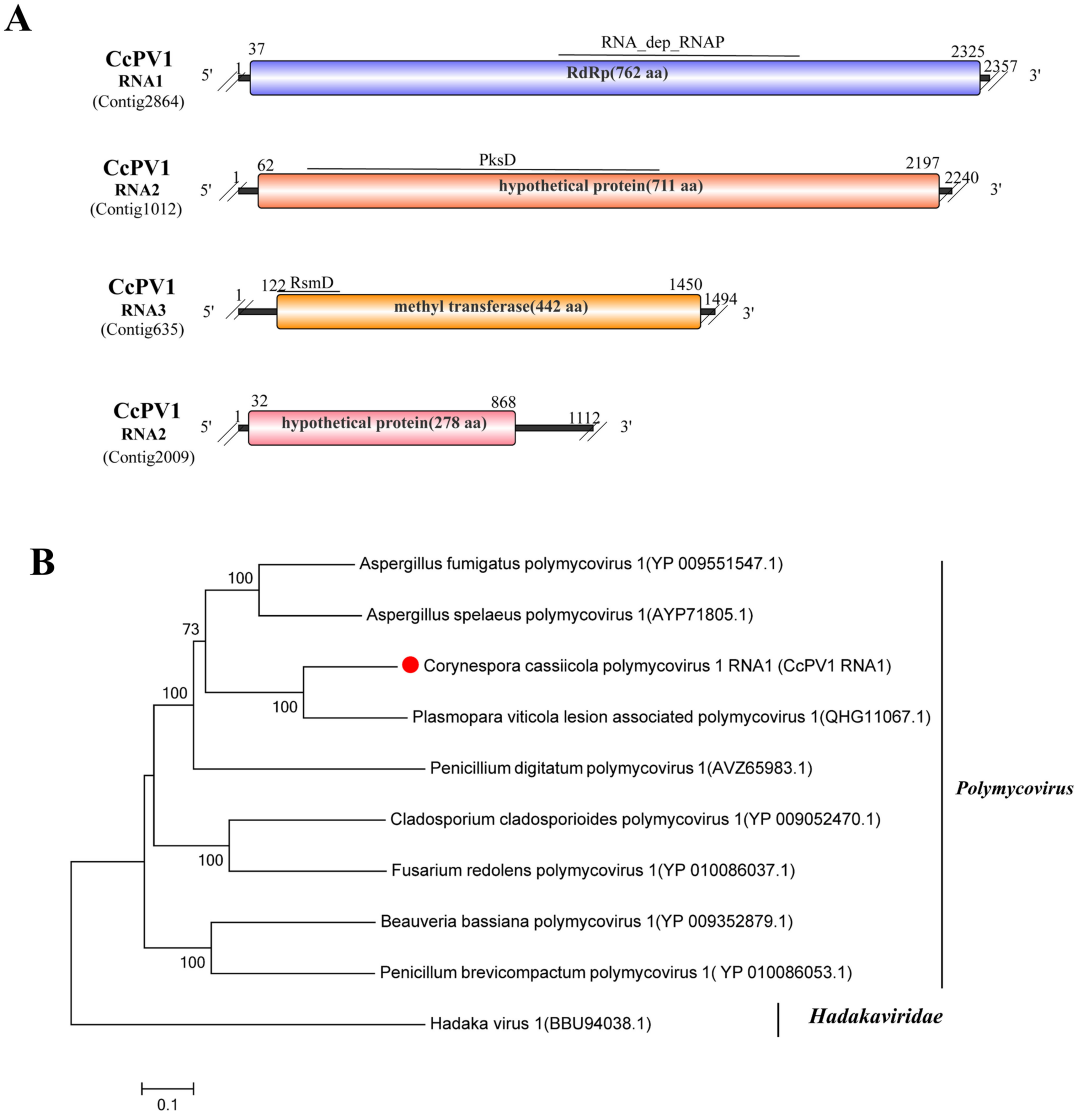
Characterization of the genome structure and phylogenetic analysis of viruses belonging to the *Polymycoviridae* family. **(A)** Schematic representation of the genome organization of viruses belonging to the *Polymycoviridae* family. ORFs are indicated by rectangular boxes. Double slashes indicate incomplete genome nucleotide sequences. **(B)** A phylogenetic analysis of the putative viruses in the family *Polymycoviridae* was performed. The amino acid sequences of the RdRp proteins were aligned using CLUSTAL X, and the phylogenetic tree was constructed with the neighbor-joining (NJ) method. The bootstrap consensus tree (1,000 replicates) is shown, with values ≥ 70% displayed at the branch nodes. Branch lengths are proportional to the number of amino acid substitutions and are measured by a scale bar. CcPV1 is indicated in the phylogenetic tree.

Genomic structure analysis showed the following: CcPV1 RNA1 encodes RdRp with the conserved RNA_dep_RNAP (cd01699) domain. CcPV1 RNA2 encodes a putative PksD protein (CL43841). CcPV1 RNA3 encodes a methyltransferase (RsmD; COG0742). CcPV1 RNA4 encodes a hypothetical protein (**Figure 12A**). Phylogenetic analysis based on the amino acid sequence of the CcPV1 RNA1-encoded polypeptide placed CcPV1 within a clade containing members of the genus *Polymycovirus* with high bootstrap support (**Figure 12B**).

Based on its distinct genomic organization and phylogenetic position that meet the ICTV species demarcation criteria for the genus *Polymycovirus*, CcPV1 is proposed as a novel member of the family *Polymycoviridae*. This taxonomic proposal is supported by following key lines of evidence: its initial identification in a strain of *C. cassiicola*, possession of the characteristic four dsRNA segments, and an RdRp amino acid sequence exhibiting less than 70% identity to the known polymycoviruses.

## 4 Discussion

In recent decades, the rapid development of high-throughput sequencing and bioinformatics technology has driven significant progress in metagenomics, leading to a dramatic expansion of the known viral sequence space. Viromics, the study of viral communities through metagenomic approaches, has been widely applied to investigate viruses in diverse environments, including fungi (Picarelli et al., 2019), soil (Chen et al., 2022), marine ecosystems (Breitbart et al., 2002), and feces (Blinkova et al., 2010). This approach enables not only the rapid acquisition of multiple viral sequences but also the detection of low-abundance viruses. Consequently, deep sequencing is now extensively used for novel virus discovery in fungi. For example, Marzano et al. employed high-throughput sequencing to analyze five widely distributed plant pathogenic fungi (*Colletotrichum truncatum*, *Macrophomina phaseolina*, *Diaporthe longicolla*, *Rhizoctonia solani*, and *Sclerotinia sclerotiorum*), identifying 66 previously undescribed fungal viruses (Marzano et al., 2016). Wang et al. characterized 27 fungal viruses in the mango leaf spot pathogen (Wang et al., 2025a). Bartholomäus et al. detected 17 distinct fungal viruses in *Rhizoctonia solani* using deep sequencing (Bartholomäus et al., 2016). Li et al. revealed an extraordinary diversity of fungal viruses in the wheat sharp eyespot pathogen (*Rhizoctonia cerealis*) through high-throughput sequencing (Li et al., 2023).

In this study, we performed metatranscriptomic sequencing of seven *Corynespora* strains isolated from sesame and identified a total of 25 mycoviral contigs representing 19 distinct viruses belonging to 12 families. The majority (13) were +ssRNA viruses, followed by three −ssRNA viruses and four dsRNA viruses. The +ssRNA viruses were assigned to the families *Botourmiaviridae* (4), *Deltaflexiviridae* (1), *Fusariviridae* (2), *Narnaviridae* (2), Ambiguiviridae (2), and *Potyviridae* (1). The −ssRNA viruses belonged to *Mymonaviridae* (2) and Mycophioviridae (1). The dsRNA viruses were classified within *Chrysovirida*e (1), *Partitiviridae* (1), *Totiviridae* (1), and *Polymycoviridae* (1). Except for CcVV1 and CcFV1, which we previously reported, the remaining viruses represent newly discovered mycoviruses in *C. cassiicola.* These findings reveal a diverse community of RNA viruses associated with *C. cassiicola* strains and provide a foundation for investigating the origin and evolution of fungal viruses. It should be noted, however, that the phylogenetic trees for these identified viruses were constructed using only the neighbor-joining method, without maximum likelihood or Bayesian inference methods, and this limitation may affect the statistical support for the inferred phylogenetic relationships.

To verify the authenticity of the obtained viral sequences, we designed specific primers for each contig and performed RT-PCR to confirm the presence and number of viruses in each of the seven *C. cassiicola* strains (**Supplementary Table 3**). Strain N3 carried the highest number of viral contigs (nine), and four strains carried six viral contigs each, whereas strain N6 contained the fewest (only one viral contig). These results demonstrate that every strain is coinfected by multiple viruses belonging to different families, a situation also reported in other mycovirus studies (Bartholomäus et al., 2016; Li et al., 2023). Such multi-viral infections underscore the ubiquity of mycoviruses and the extraordinary diversity within *C. cassiicola*, providing a valuable resource for investigating fungus–virus interactions.

With advancing understanding of the biological characteristics and molecular features of mycoviruses, their multifaceted roles in fungal ecology are receiving growing recognition. Recent studies have identified hypovirulence-associated mycoviruses in multiple fungal species, including *Sclerotinia sclerotiorum* (Ye et al., 2025), *Alternaria* species (Wang et al., 2025b), and *Colletotrichum gloeosporioides* (Hassan et al., 2025). Notably, the chrysovirus CcChv1 was detected in strain N3, which exhibited slow growth and attenuated virulence. This correlation suggests that CcChv1 may affect the fungal host’s development and pathogenicity. Our hypothesis is supported by previous reports on related chrysoviruses. For example, MoCV1-A (Urayama et al., 2010), MoCV1-B (Urayama et al., 2014), and MoCV1-D (Higashiura et al., 2019) induce hypovirulence-associated phenotypes in the rice blast fungus—including reduced growth, pigment accumulation, and abnormal hyphal morphology—similar to those observed in strain N3. Similarly, various chrysoviruses identified in *Fusarium* species can alter colonial morphology and reduce pathogenicity (Zou et al., 2024). In future studies, we will employ curing and transfection experiments to verify the specific role of CcChv1 in regulating the pathogenicity and morphology of *C. cassiicola*. These studies will also help evaluate the potential of CcChv1 in biocontrol applications. Likewise, CcPV1, assigned to the family *Polymycoviridae*, may be responsible for the morphological anomalies seen in strain N2. Several reports have documented that strains infected by polymycoviruses exhibit pigment changes (Filippou et al., 2021), growth retardation (Zhai et al., 2016; Jia et al., 2017), and reduced virulence toward plants (Zhai et al., 2016; Jia et al., 2017). Additionally, CcPov1 may be the *Potyviridae*-like contig, which may originate from plant RNA, and therefore requires validation to confirm its identity as a mycovirus. However, strain N6 also shows slow growth and abnormal colony morphology, yet only one viral sequence was detected; this discrepancy could be due to insufficient sequencing depth or the presence of undetected DNA viruses. The putative hypovirulence-associated mycoviruses identified here represent promising candidates for future biological control of sesame leaf spot disease.

## 5 Conclusions

In this study, we employed high-throughput sequencing to detect mycoviruses in seven strains of *C. cassiicola* isolated from sesame. We identified a total of 19 mycoviruses, of which 12 were +ssRNA viruses, 3 were -ssRNA viruses, and 4 were dsRNA viruses. Sequence alignment and phylogenetic analysis indicated that these viral sequences could be assigned to 12 established or putative virus families: *Botourmiaviridae*, *Deltaflexiviridae*, *Fusariviridae*, *Narnaviridae*, Ambiguiviridae, *Potyviridae*, *Mymonaviridae*, Mycophioviridae, *Chrysoviridae*, *Partitiviridae*, *Totiviridae*, and *Polymycoviridae*. RT-PCR validation confirmed that each tested strain harbored mycoviruses, with many exhibiting coinfection by multiple viruses. This reveals a high prevalence of mycoviruses in *C. cassiicola* with diverse infection patterns. The discovery of these novel viruses in the *C. cassiicola* significantly expands our understanding of fungal virus diversity and provides new perspectives for investigating the evolutionary dynamics and potential ecological functions of viruses within their fungal hosts.

## Data Availability

The datasets presented in this study can be found in online repositories. The names of the repository/repositories and accession number(s) can be found in the article/**Supplementary Material**.

## Supplementary material

Supplementary Figure 1 Virulence of Strains N4 and N3 on detached sesame leaves at 6 days after Inoculation.

Supplementary Figure 2 RT-PCR electrophoresis detection of some viral genome segment in 7 C*. cassiicola* strains. Abbreviates of viruses are on the right side of the lane. Lane M, DL2000 DNA Marker (Takara Bio Inc., Japan).

Supplementary Figure 3 Type and proportion of the mycoviruses. **(A)** The quantity and proportion of viruses of different genotypes. **(B)**Virus classification status and proportion at the family level.

Supplementary Figure 4 Motif C of RdRp from CcAV1 and CcAV2 and related viruses. CcAV1 and CcAV2 contains the GDN triad in Motif of the RdRp, while canonical GDD motif found in *Tombusviridae*.
