# Supplementary figures and images for: Diverse, novel mycoviruses coinfecting the phytopathogenic fungus Corynespora cassiicola from Sesamum indicum
Source: Front Cell Infect Microbiol. 2026 Jan 5;15:1704628. doi: 10.3389/fcimb.2025.1704628 (PMC12812968; doi:10.3389/fcimb.2025.1704628)

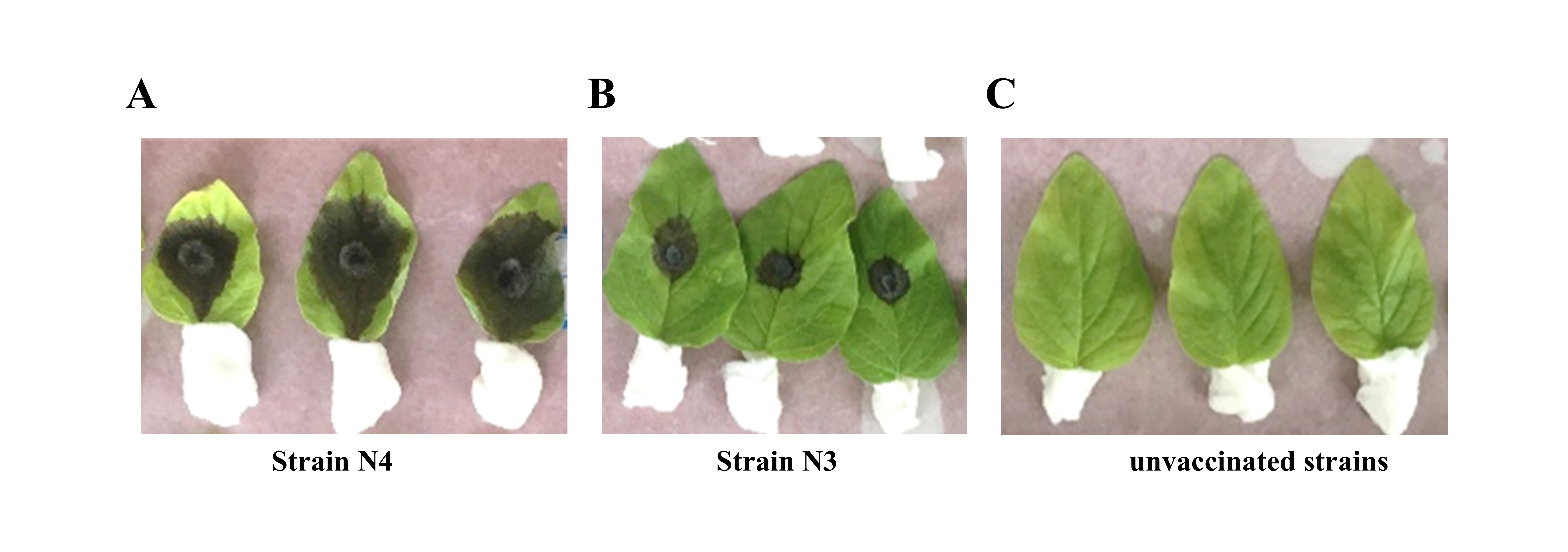

Supplement: Supplementary Figure 1 — Virulence of Strains N4 and N3 on detached sesame leaves at 6 days after Inoculation. [file Image1.tif]

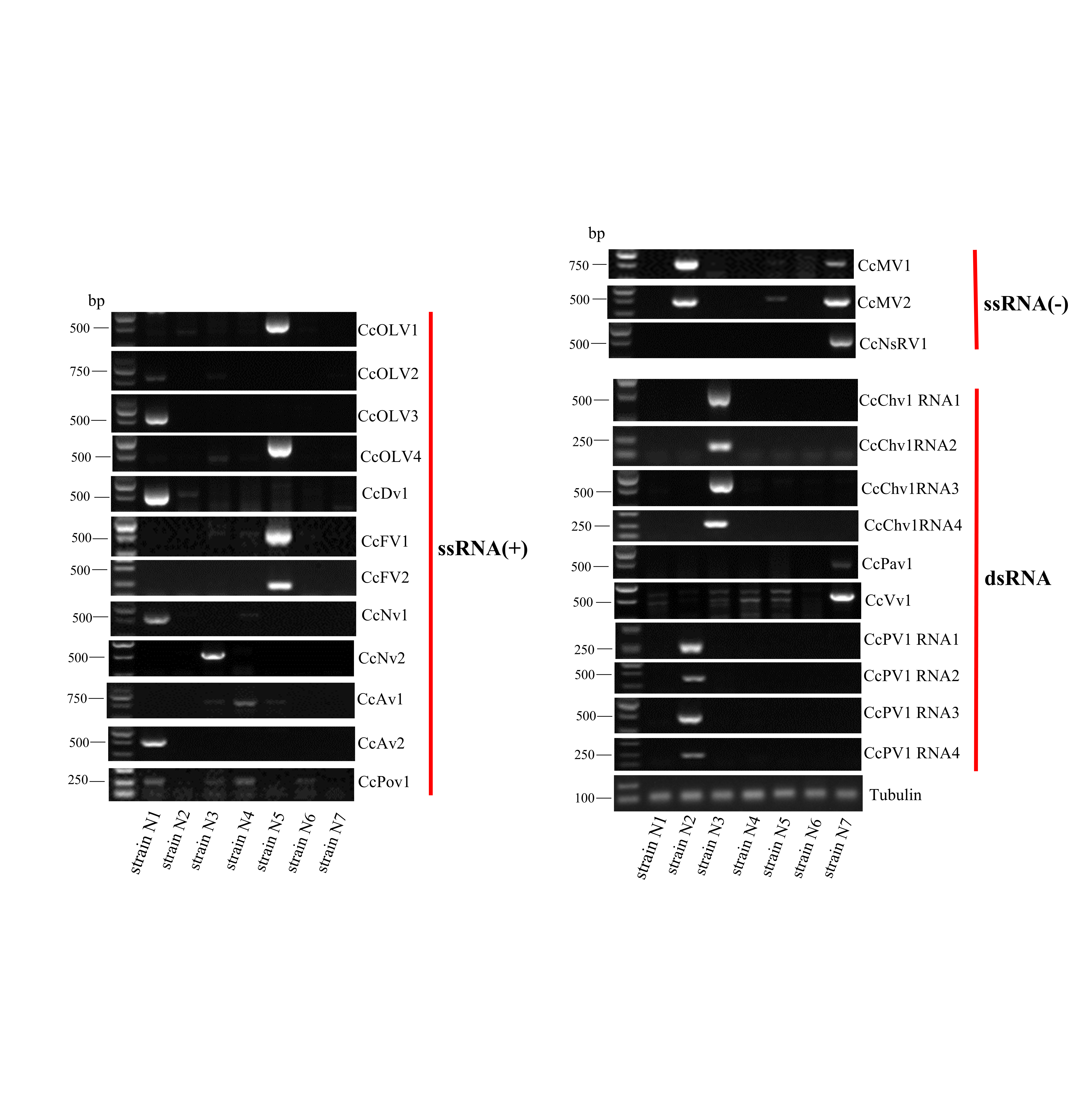

Supplement: Supplementary Figure 2 — RT-PCR electrophoresis detection of some viral genome segment in 7 C. cassiicola strains. Abbreviates of viruses are on the right side of the lane. Lane M, DL2000 DNA Marker (Takara Bio Inc., Japan). [file Image2.tif]

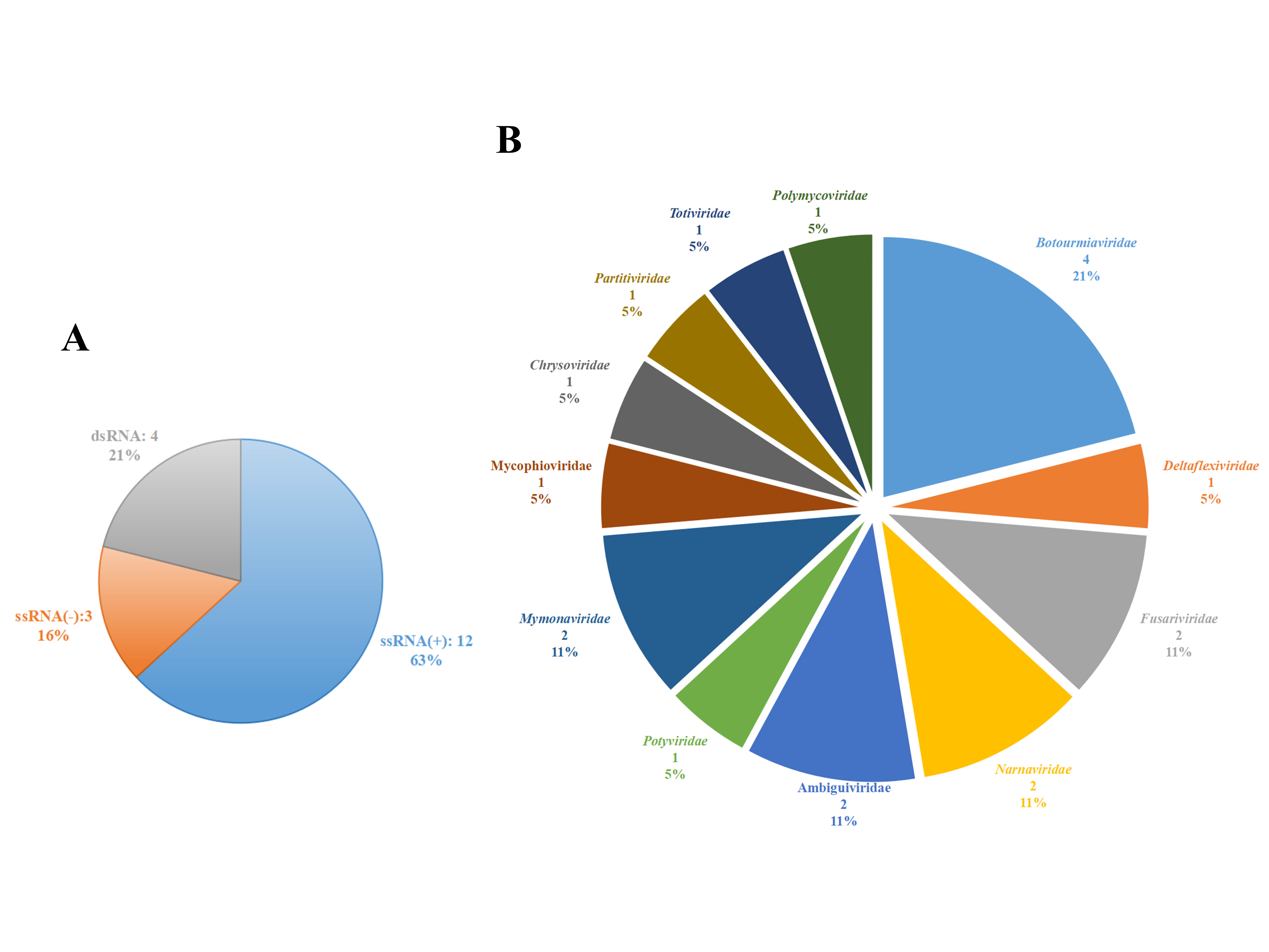

Supplement: Supplementary Figure 3 — Type and proportion of the mycoviruses. (A) The quantity and proportion of viruses of different genotypes. (B)Virus classification status and proportion at the family level. [file Image3.tif]

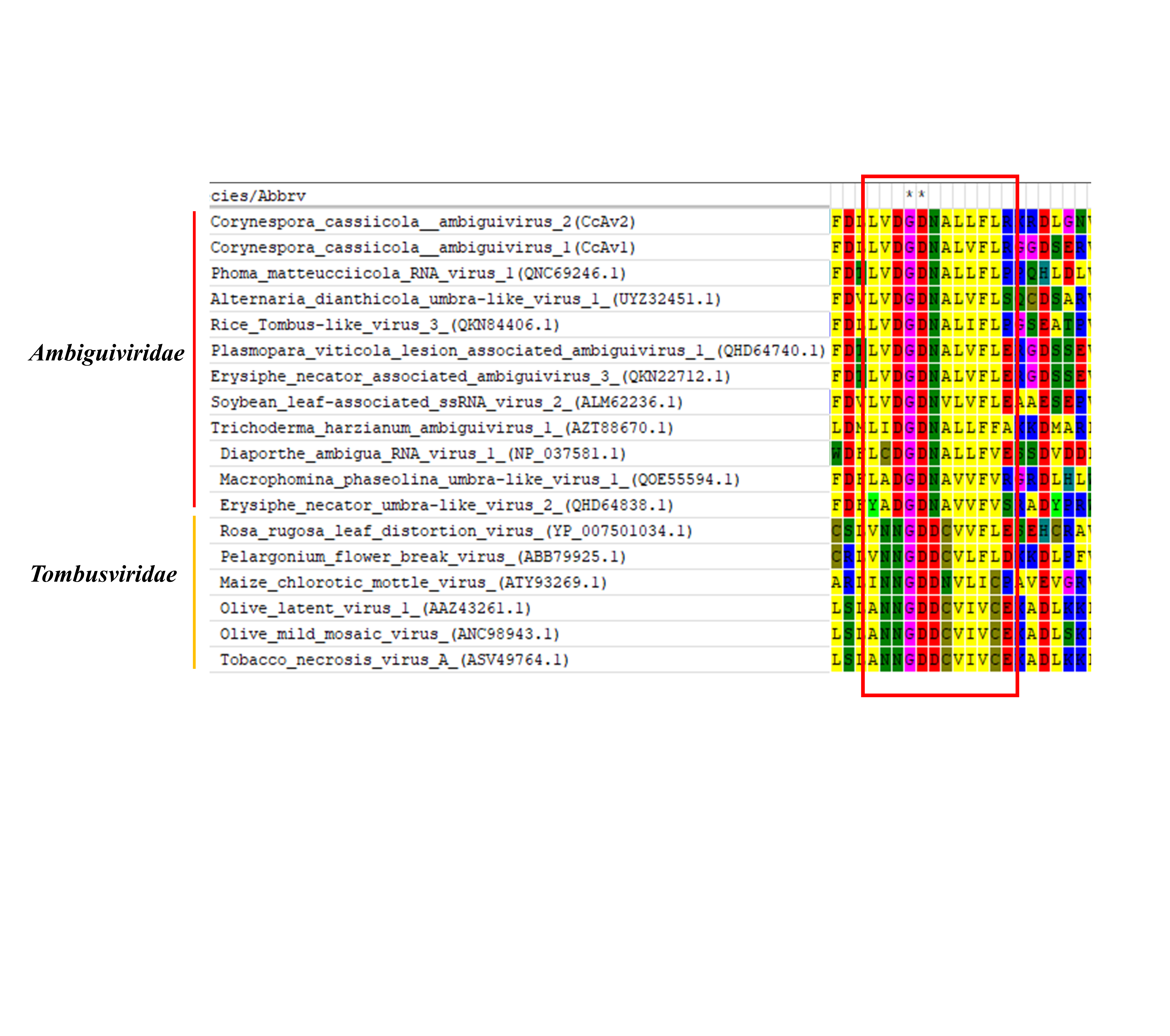

Supplement: Supplementary Figure 4 — Motif C of RdRp from CcAV1 and CcAV2 and related viruses. CcAV1 and CcAV2 contains the GDN triad in Motif of the RdRp, while canonical GDD motif found in Tombusviridae. [file Image4.tif]

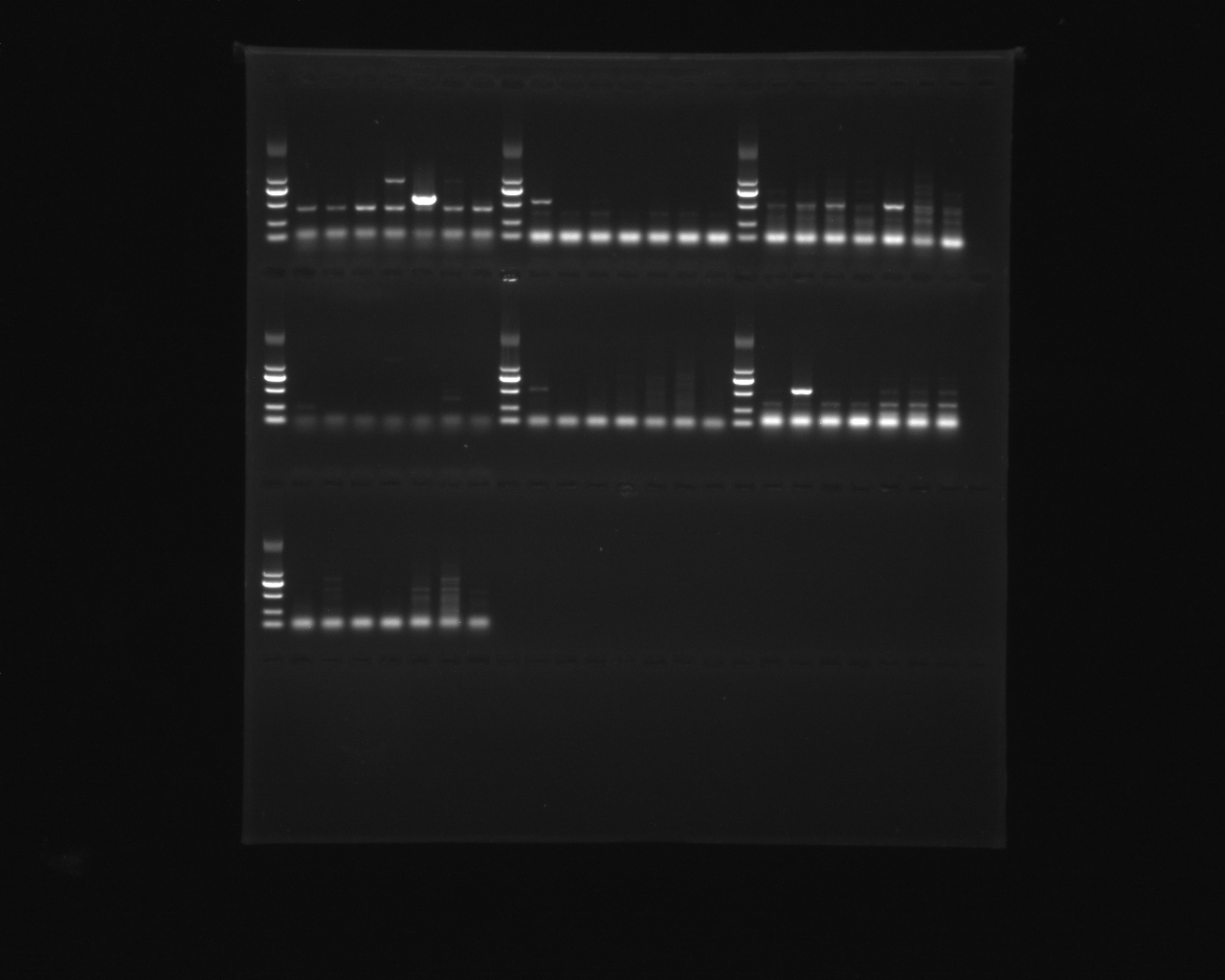

Supplement: Supplementary file 6 [file DataSheet1.zip › 1.AContig181_Contig185_Contig1442_AFirstContig281_Contig1327_Contig27_AContig1246.Tif]

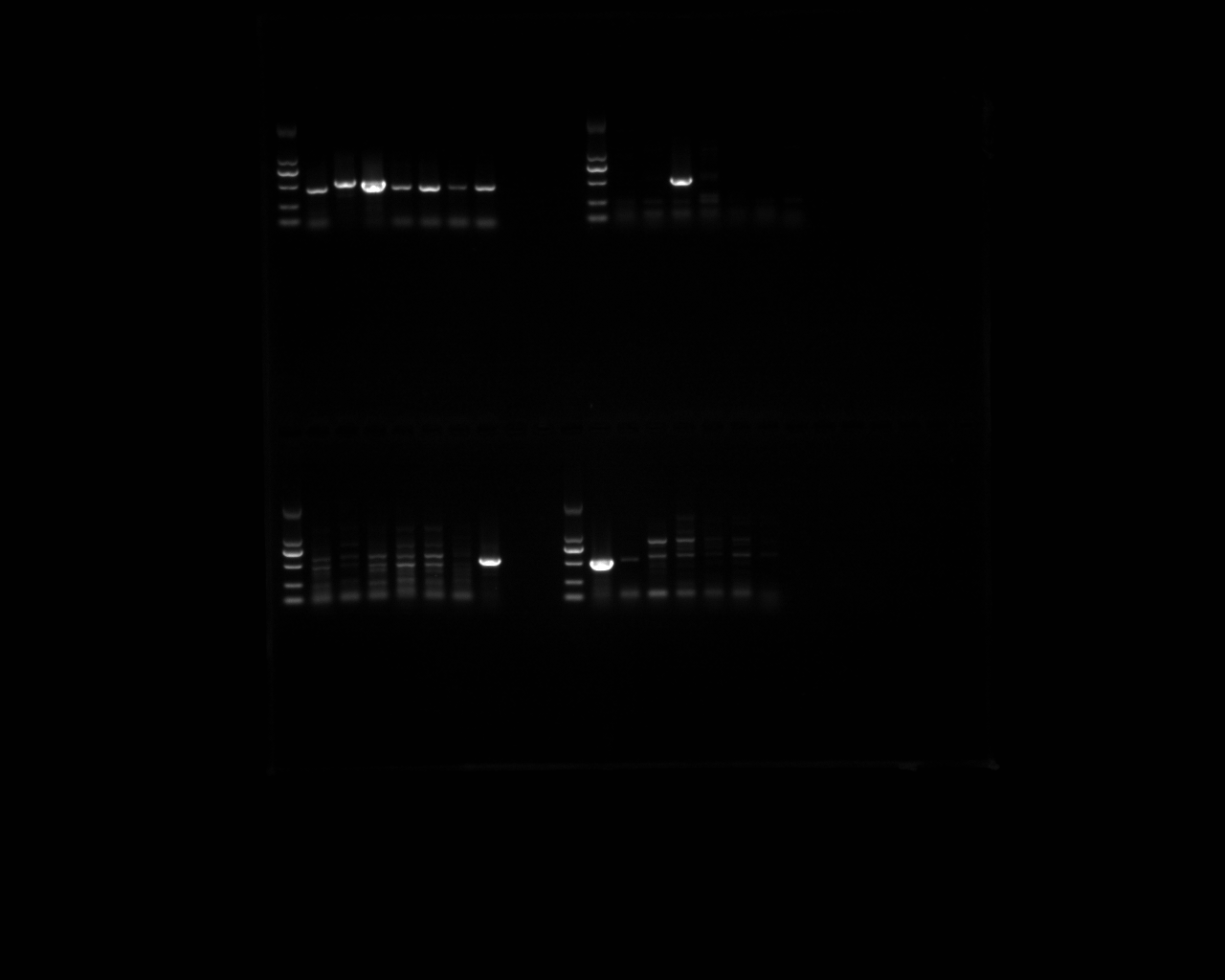

Supplement: Supplementary file 6 [file DataSheet1.zip › 2.Contig885_Contig1035_Contig1246_Contig1327.Tif]

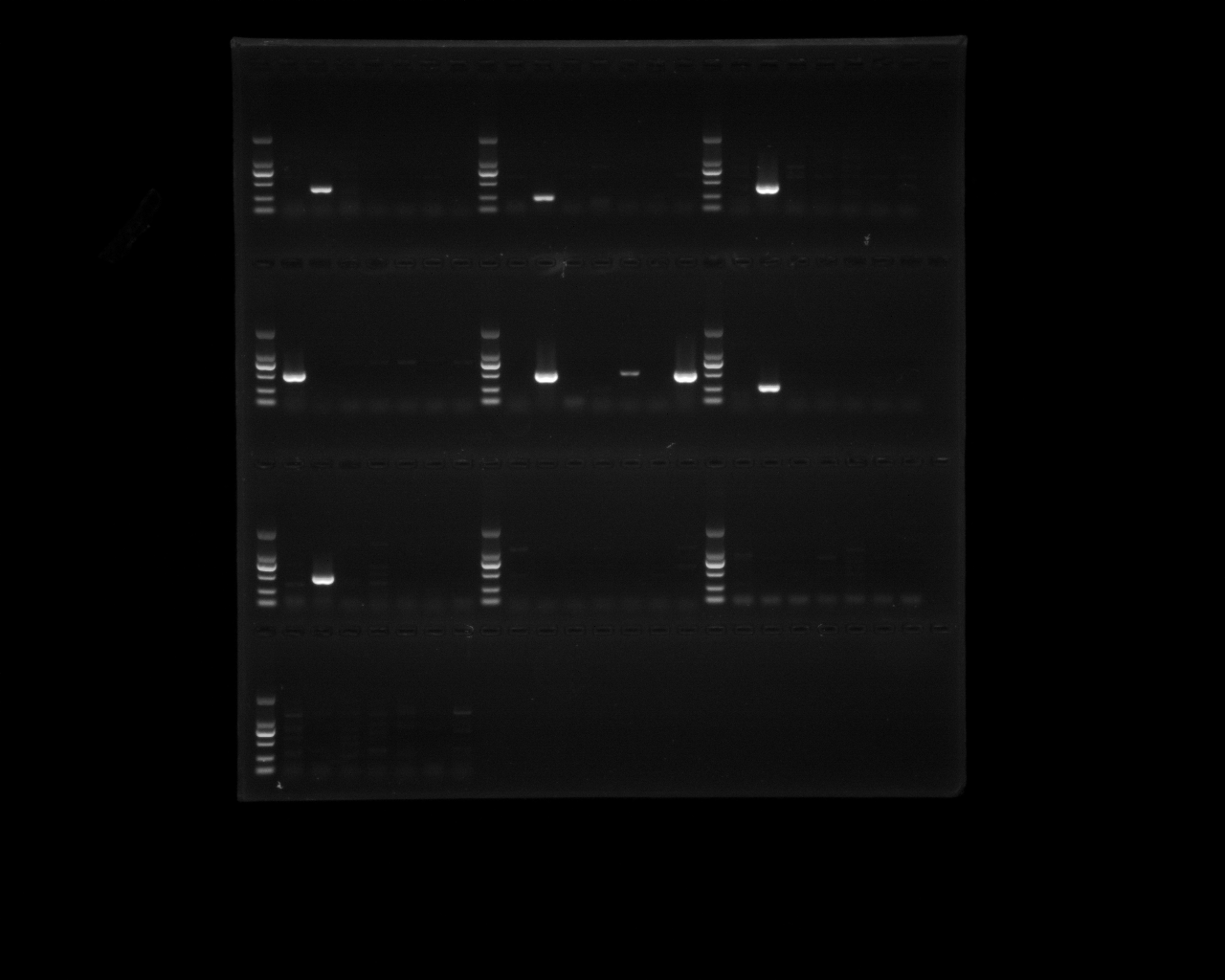

Supplement: Supplementary file 6 [file DataSheet1.zip › 3.Contig1012_Contig2009_Contig98_Contig333_Contig28_Contig2864_Contig635_Contig424_Contig11936_Contig14603.Tif]

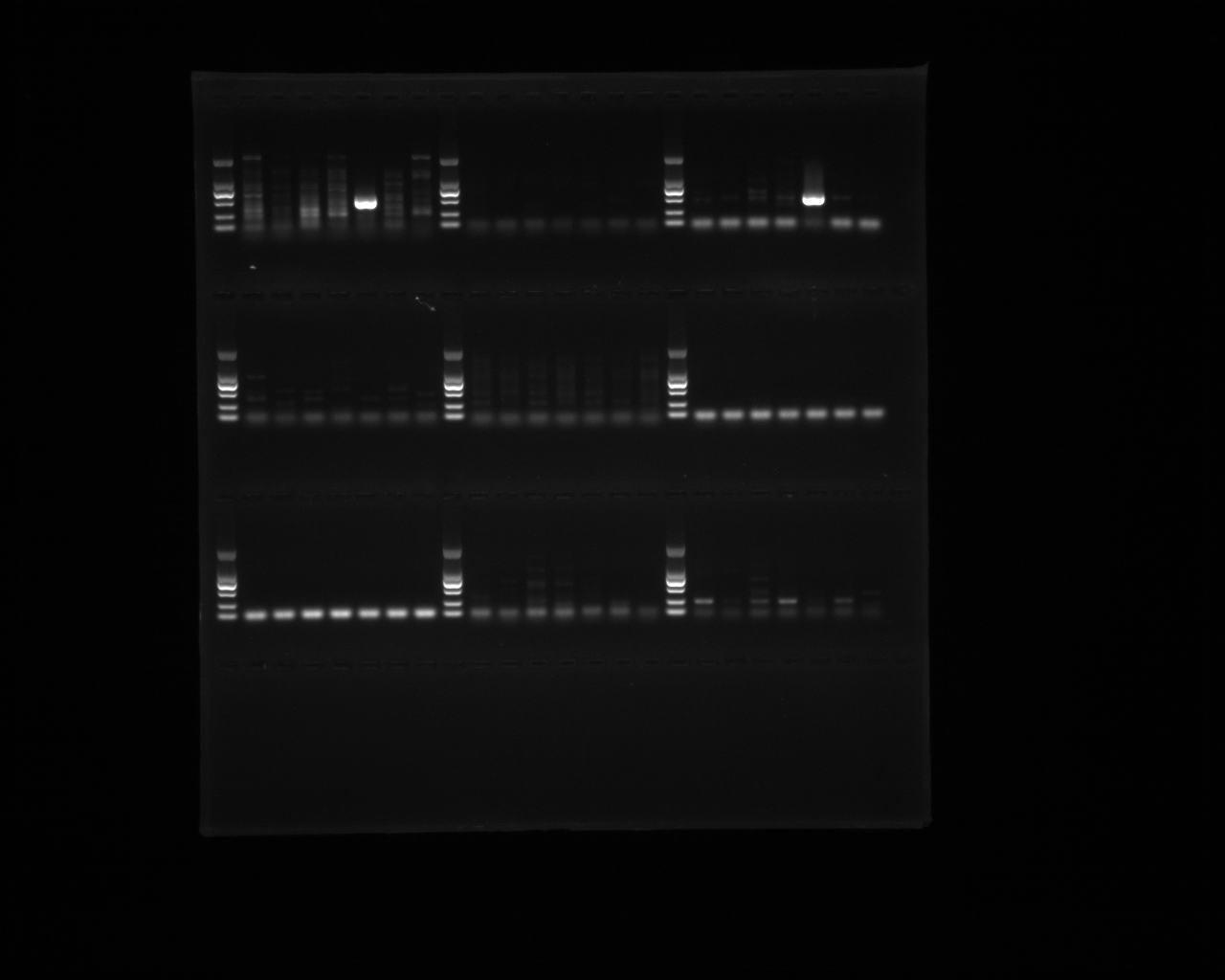

Supplement: Supplementary file 6 [file DataSheet1.zip › 4.Contig1442_Contig2181_Contig2308_Contig7789_Contig8337_Contig12007_Contig12039_Joined-Contig1-2_FirstContig281.Tif]

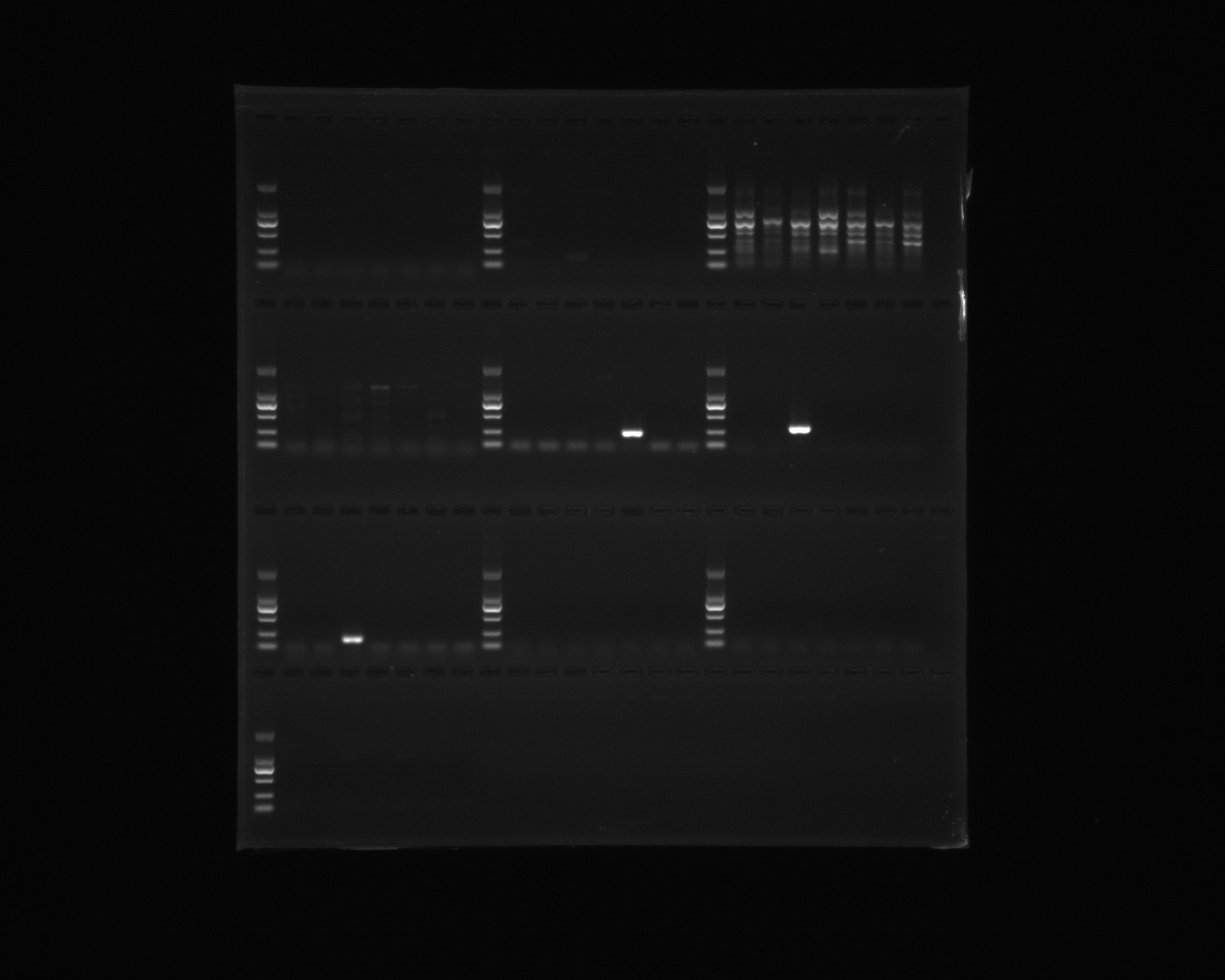

Supplement: Supplementary file 6 [file DataSheet1.zip › 5.Contig13503_Contig13026_Contig15557_Contig10995_Contig3361_Contig157_Contig30_Contig30485_Contig229_Contig4431.Tif]

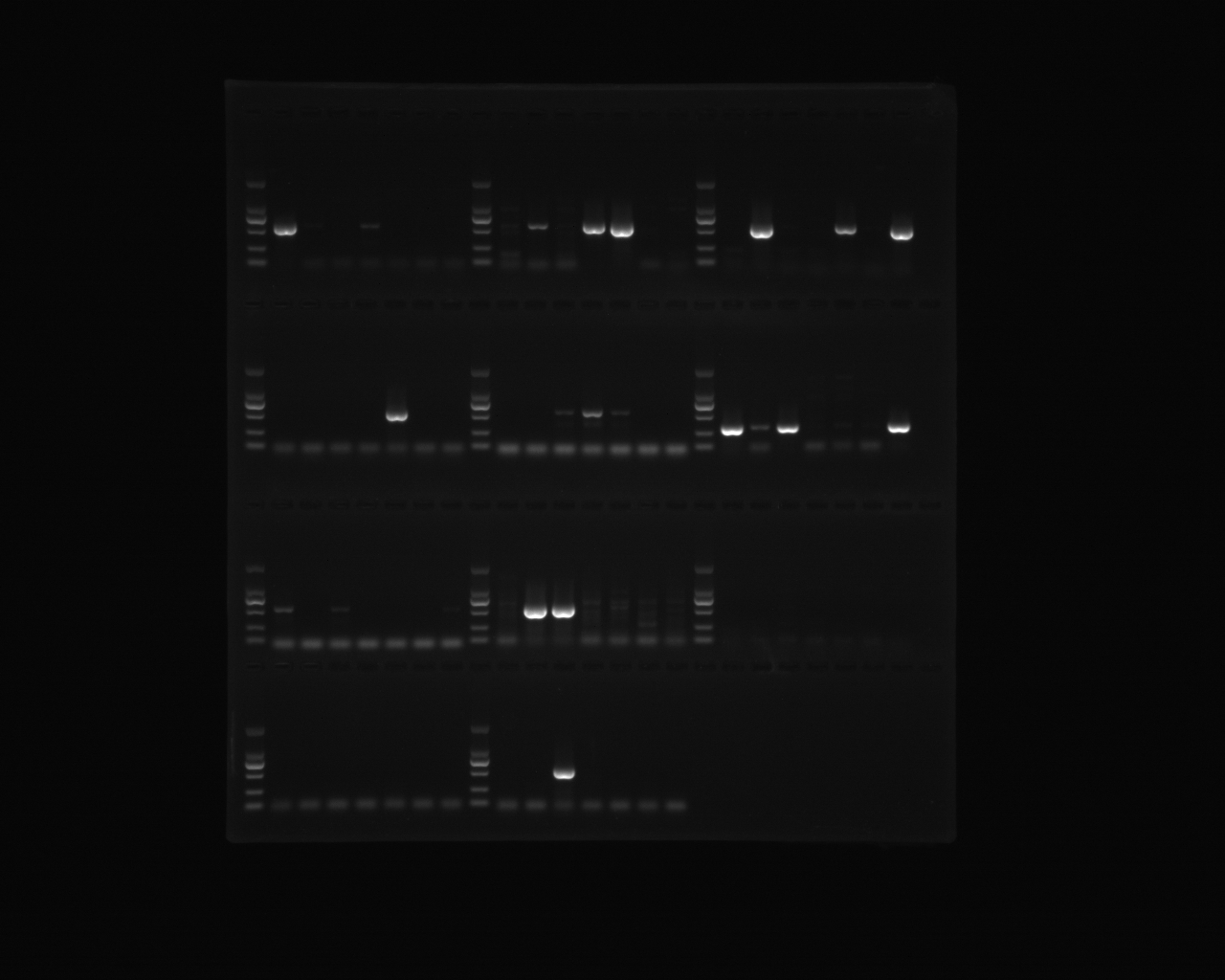

Supplement: Supplementary file 6 [file DataSheet1.zip › 6.FirstContig1_FirstContig2_FirstContig7_FirstContig10_FirstContig11_FirstContig24_FirstContig27_FirstContig39_FirstContig49_FirstContig75_FirstContig69.Tif]

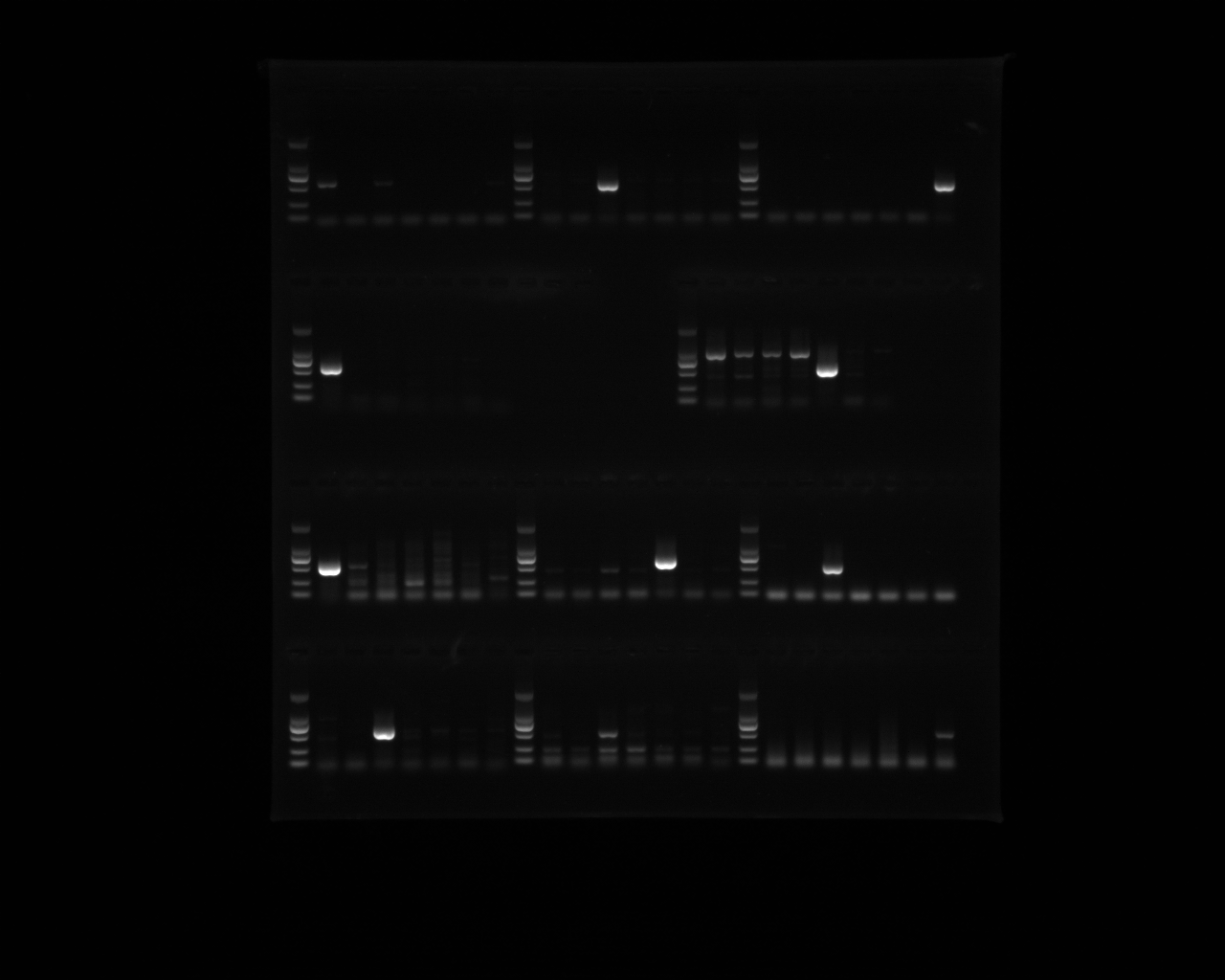

Supplement: Supplementary file 6 [file DataSheet1.zip › 7.FirstContig27_FirstContig69_Contig47_Contig163_Contig181_Contig185_Contig532_Contig829_Contig895_Contig1035_Contig1249.Tif]

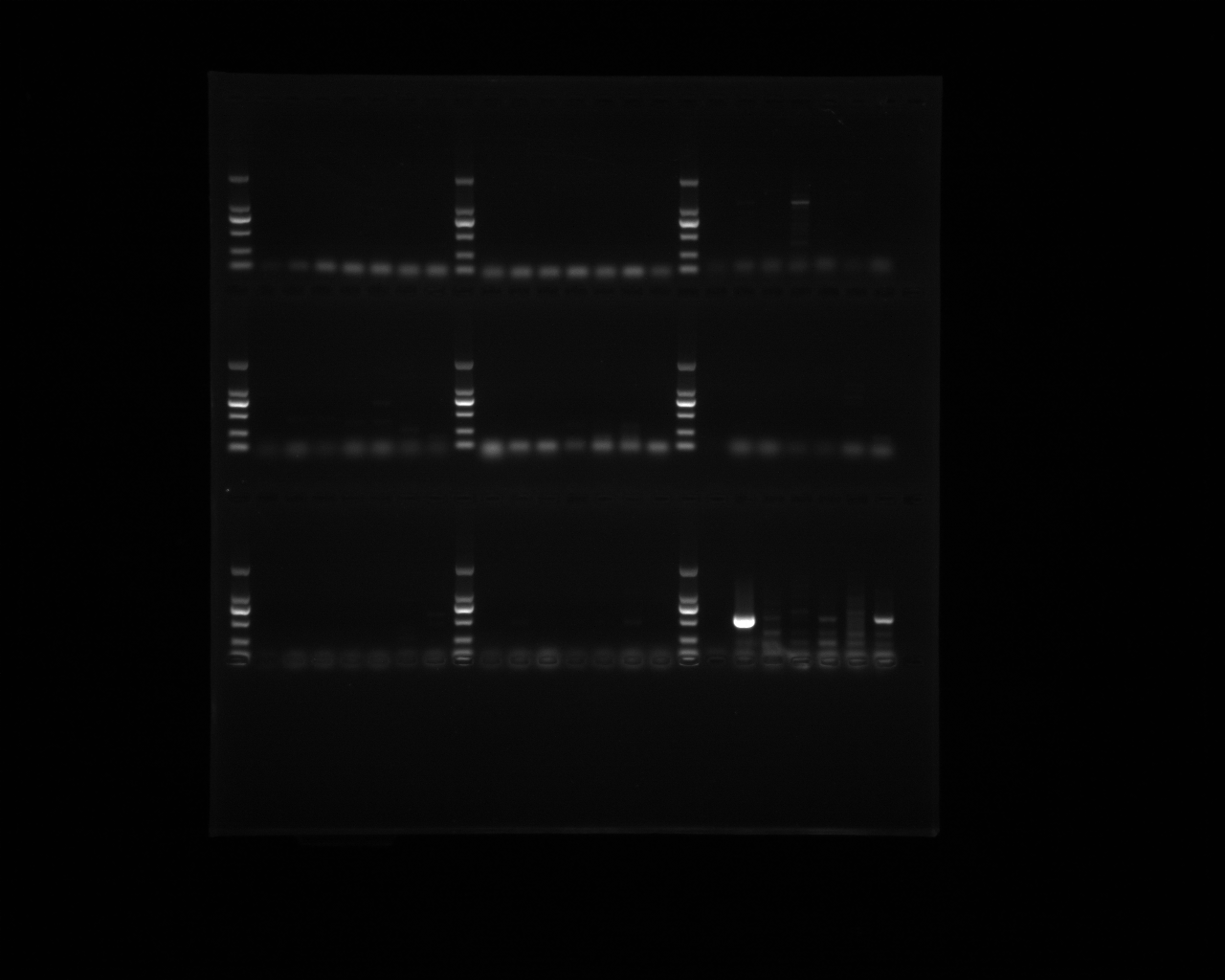

Supplement: Supplementary file 6 [file DataSheet1.zip › 8.AFirstContig281_FirstContig286_FirstContig619_FirstContig701_FirstContig833_FirstContig850_FirstContig1254_FirstContig1474_Contig27.Tif]

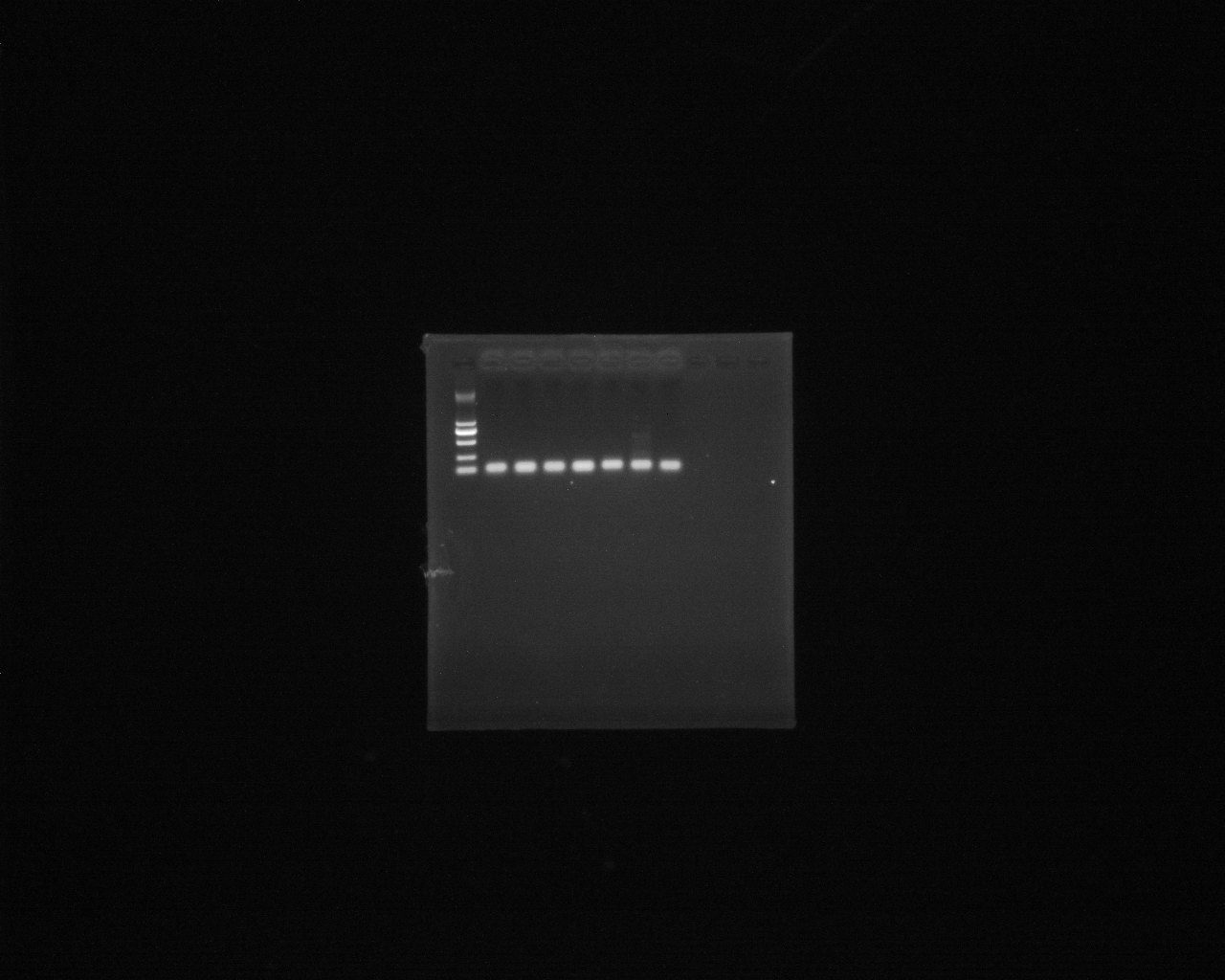

Supplement: Supplementary file 6 [file DataSheet1.zip › 9.tublin.Tif]
